# Acknowledgment to the Reviewers of *Plants* in 2022

**DOI:** 10.3390/plants12030573

**Published:** 2023-01-28

**Authors:** Plants Editorial Office

**Affiliations:** MDPI AG, St. Alban-Anlage 66, 4052 Basel, Switzerland

High-quality academic publishing is built on rigorous peer review. *Plants* was able to uphold its high standards for published papers due to the outstanding efforts of our reviewers. Thanks to the efforts of our reviewers in 2022, the median time to first decision was 14 days and the median time to publication was 36 days. Regardless of whether the articles they examined were ultimately published, the editors would like to express their appreciation and thank the following reviewers for the time and dedication that they have shown *Plants*:

Abakumov, Evgeny V.Llorente, IsidreAbate, GiuliaLloret, AlbaAbate, MarioLlugany, MercèAbayomi-Alli, Olusola OluwakemiLobato, Allan Klynger Da SilvaAbbai, RagavendranLocci, FedericaAbbasi Moud, ArefLoeza-Lara, PedroAbbate, LoredanaLöhrer, MarcoAbbey, LordLohwasser, UlrikeAbd El-Mageed, TaiaLoizzo, Monica R.Abdallah, MohamedLombardo, LucaAbdel Latef, Arafat Abdel HamedLomonossoff, George P.Abdelaal, KhaledLomovatskaya, LidiaAbdel-Aziz, Heba Mahmoud MohammedLone, Ajaz A.Abd-ElGawad, Ahmed M.Long, YuchenAbdelraheem, AbdelraheemLopes, GracilianaAbd-Elsalam, KamelLopes-Caitar, Valéria StefaniaAbdulwahab, Shadma W.López Ocaña, Víctor CicuéndezAbdur, Rashid MohammadLópez Solanilla, EmiliaAbedi, TayebehLópez, María D.Abiri, RambodLópez-Colom, PaolaAbo-Elyousr, Kamal A. M.López-Cristoffanini, CamiloAbou Fayssal, SamiLópez-Gallego, ElenaAbraham, EleniLopez-Marin, JosefaAbugri, Daniel A.Lopez-Meyer, MelinaAcevedo, Juan JoseLópez-Rosas, HugoAcquaviva, RosariaLoppi, StefanoAdadi, PariseLops, FrancescoAdamakis, Ioannis-DimosthenisLora, JorgeAdamczewska-Sowińska, KatarzynaLorenz, PeterAdamczyk, GretaLorenzo, PaulaAdamec, LubomírLorite, MariaAdamec, ZdeněkLošák, TomášAdamiec, MałgorzataLöschenberger, FranziskaAdamski, ZbigniewLotanna Micah, NnejiAdamson, KalevŁotocka, BarbaraAdil, MuhammadLou, YanhongAdkar-Purushothama, Charith RajLøvdal, TrondAdnan, MdLow, Yee WenAdnan, MohdLoziene, KristinaAdnan, MuhammadLstibůrek, MilanAeron, AbhinavLu, GuodongAfanasenko, Olga SilvestrovnaLu, HaiweiAfonin, AlexeyLu, Mei-ChinAfonnikov, Dimitry ArkadievichLu, MengmengAgarwala, NirajLu, Pei-LuenAgorio, AstridLu, QingtaoAgrawal, MadhoolikaLu, YanhuiAgrawal, ShilpiLu, YouAgriopoulou, SofiaLuca, Simon VladAguilos, MaricarLucardi, RimaAguirre, CarlosLucarini, MassimoAgyin-Birikorang, SampsonLuci, GiacomoAhmad, MuhammadLucini, LuigiAhmed, BilalLuciński, RobertAhmed, MukhtarLucioli, SimonaAhmed, NiazLücking, RobertAhmed, Zienab F. RLuczaj, LukaszAhn, Mi-JeongLudidi, NdikoAigotti, RiccardoLuebken, TiloAinane, TarikLuigi, MartaAioub, AhmedLuis, Jorge C.Ait-El-Mokhtar, MohamedLuisa Buide, MariaAkhmanova, MariaŁukaszewska, AleksandraAkhter, FirozLukaszewski, Adam J.Akimoto, MasahiroLukatkin, Lukatkin, Alexander S.Akiyama, MasahiroLukić, IgorAkkak, AzizLukinac Čačić, JasminaAkmal, MohammadLukinich-Gruia, Alexandra TeodoraAksmann, AnnaŁukowski, AdamAkter, AyashaŁukowski, AdrianAl Tawah, Abdel RahmanLuliński, PiotrAlam, AfrozLun, GaoAlam, PravejLuna, Ana P.Alam, PrawezLuna-Arias, Juan PedroAlam, RejbanaLundgren, MarjorieAlamillo, Josefa MuñozLung, Ming-YuAl-Amri, Issa SulaimanLungu, MihaelaAlatzas, AnastasiosLuo, ChuanxiuAlbert, BeatriceLuo, PeigaoAlberto Marco, Del PinoLuo, WeiAlbreht, AlenLupp, GerdAlcaide, CristinaLuque, FranciscoAlcántara-de la Cruz, RicardoLuu, Van T.Alcazar Magana, ArmandoLuvisi, AndreaAlché Ramírez, Juan De DiosLyanguzova, IrinaAlcock, ThomasŁyczakowski, JanAldrete, ArnulfoŁyczko, JacekAleksandar, CingelLynch, Joseph H.Aleksandrov, VladimirŁysiak, GrzegorzAleksashin, NikolayLystvan, KaterynaAlekseenko, AlexeyLyu, Jae IlAlemu, AdmasLyubomirova, ValentinaAlessandra, BonettiM. Bastías, RichardAlex, AnoopMa, GuohuaAlexa, ErsiliaMa, LitingAlexander, GulevichMa, TaoAlexandris, StavrosMa, XueyingAlexandrov, Oleg SergeevichMa, YifanAlexiou, KonstantinosMa, YubinAlexoaei, Alina PetronelaMaalouf, Fouad S.Alexopoulos, Alexios A.Macák, MilanAlexopoulos, AthanassiosMaçãs, BenvindoAleynova, Olga A.Macchioni, NicolaAlfei, SilvanaMaćešić, DubravkoAlfonso, MiguelMachado, RodrigoAli Ahmed, Esmat FaroukMachado, Silvia RodriguesAli Raza, MuhammadMacía Barco, Manuel JuaAli, AkhtarMaciej, StrzemskiAli, AshaqMacioszek, ViolettaAli, EsmatMacolino, StefanoAli, Muhammad AmjadMacrì, RobertaAli, QurbanMadalina, IugaAli, SajadMaddalena, GiulianaAli, SajidMaděra, PetrAli, ShaidMaduna, SimoAlia-Tejacal, IranMaduraimuthu, DjanaguiramanAlipieva, KalinaMaekawa, ShugoAlishetty, SumanMafrica, RoccoAllaby, RobinMaggert, KeithAllan, Gerard J.Maggio, AlbinoAllevato, EnricaMagnano Di San Lio, GaetanoAl-Maharik, NawafMago, RohitAlmutairi, Khalid F.Magri, DonatellaAlok, AnshuMagrini, SaraAlonso-Castro, Angel JosabadMaguilla, EnriqueAlpuerto, Jasper Benedict B.Maharachchikumbura, SajeewaAlsina, InaMahmood, IrshadAl-Taey, Duraid K. A.Mahmood, QaisarAltieri, SimonaMahmoud, Eman E.Altland, JamesMahmoudi, NiloufarÁlvarez Fernández, InésMahmudul Huque, A. K. M.Alvarez, AlejandraMahomoodal, FawziAlvarez, RobertoMaicas, SergiÁlvarez-Maldini, CarolinaMaier, CameliaAlvarez-Suarez, JoseMaietti, AnnalisaAlvear, MarysolMaitra, PulakAlves, EduardoMajchrzak, TomaszAlves, Marcelo de CarvalhoMajewska, MałgorzataAlves, SheilaMajewski, EdwardAlwarthan, AbdulrahmanMajidi, LhouAl-Yahyai, RashidMajláth, ImreAlzubaidi, LaithMajor, Ian T.Amadeu, RodrigoMakádi, MariannaAmaki, WakanoriMakara, AgnieszkaAmara, AmroMakarenko, Maxim StanislavovichAmarowicz, RyszardMakareviciene, VioletaAmbrožič-Dolinšek, JanaMakaroff, ChristopherAmichot, MarcelMäkinen, HarriAmidžić Klarić, DanielaMäkinen, SariAmigo-Benavent, MiryamMakino, TomoyukiAmiri, SabaMakoś, PatrycjaAmirudeen, Abdul Kader JailaniMakowski, WojciechAmit, Kumar SinghMakra, LászlóAmitrano, ChiaraMaksimov, Igor V.Ammar, El DesoukyMaksimova, Yuliya Gennad’evnaAmorim, JayrMalacarne, GiuliaAmorós, AsunciónMalaguarnera, MicheleAmpim, PeterMałajowicz, JolantaAmy Ku, Yee-ShanMalaník, MilanAna, Fernández-OcañaMaldonado-Alconada, A. M.Ana, Juan-GallardoMaleki, FarhadAnastasiou, EvangelosMaleva, MariaAnastasiu, PaulinaMalewski, TadeuszAnbazhagan, SathiyaseelanMalfa, Giuseppe AntonioAnco, Daniel J.Małgorzata, GutkowskaAncona-Contreras, VeronicaMalhó, RuiAncuceanu, RobertMalik, AnuragAndabaka, ŽeljkoMalik, Rondy JanAndersen, Mathias N.Malinowski, RyszardAnderson, AnneMalkowski, EugeniuszAnderson, JamesMallick, ShekharAndjelkovic, VioletaMallik, AzimAndrade Cetto, AdolfoMalyar, YuriyAndrade, Eugénia deMan, SimonaAndrade, GaldinoManan, SehrishAndrei, SandaManasova, MarieAndrianasolo, EricMancianti, FrancescaAndrosiuk, PiotrMandrich, LuigiAndruszczak, SylwiaMandrioli, MauroAndrzejewska, JadwigaMandzhieva, Saglara S.Aneva, Ina YosifovaManetto, GiuseppeAngelaki, AnastasiaMang, Stefania MirelaAngelini, ElisaMann, Rajinder S.Angiolella, LetiziaMannino, GiuseppeAngiolini, ClaudiaManojlović, NedeljkoAnh, La HoangManolikaki, IoannaAnisimov, Vyacheslav SergeevichManrique, EstebanAnisimova, IrinaMansoor, ShahidAnjos, OféliaMansour, ElsayedAnkica, SarajlićMansour, HaniAnnang, FrederickManyakhin, Artem YuAnsari, Mohammad AzamManzoor, HussainAnsari, PrawejManzoor, IrfanAntal, DianaMaqbool, QaisarAntille, Diogenes L.Marasco, RamonaAntognoni, FabianaMarasek-Ciolakowska, AgnieszkaAntonelli, FedericaMarček, TihanaAntón-Herrero, RafaelMarcelo, VictorianoAntoniewska, AgataMarčetić, Mirjana D.Antonkiewicz, JacekMarchioni, IlariaAntonova, DanielaMarcinkevičienė, AušraAntosik, AdrianMarcolongo, LoredanaAntunes, MauricioMarcote, María JeśusAntúnez, PabloMarcussen, ThomasAnugu, RaghunathreddyMarcysiak, KatarzynaApostolova, ElenaMarek, AdamAppendino, GiovanniMarengo, AriannaAppenroth, Klaus JuergenMaresca, VivianaAprodu, IulianaMarevci, Maša KnezAprotosoaie, Ana ClaraMarfo, Theodore DansoAqeel, MuhammadMargaoan, RodicaAramă, CorinaMargaritopoulou, TheoniAraújo, MárciaMargis, RogerioArbault, StéphaneMarhava, PetraArchacki, RafalMaria Musarella, CarmeloArduini, IdunaMaría V, RodriguezAreche, CarlosMaria-Ferreira, DanieleArellano, Juan B.Marian, EleonoraAremu, Adeyemi OladapoMariantonietta, ColagieroArévalo, José RamónMaricle, BrianArgento, SergioMarietta, FodorArgüelles, Juan CarlosMarín Sanleandro, PuraArigò, AdrianaMarinaș, IoanaArion, FelixMarino, AngelaAristizábal, Luis F.Maris, LaubertsArletti, RossellaMarius, BodorArmstrong, PaulMariychuk, RuslanArnaiz, AnaMarkham, JohnArnal Barbedo, Jayme GarciaMarkovic, DimitrijeArnaud, NicolasMarković, MonikaArnelas, ItziarMarković, TatjanaArokiyaraj, SelvarajMarković, ZvjezdanaAronen, TuijaMarkowski, MarekAronne, GiovannaMarmiroli, MartaArora, DeepikaMarmiroli, NelsonArribas, ClaudiaMarques, GuilherminaArtigas, MariaMarques, IsabelArtlip, TimothyMarques, NatáliaArtyszak, ArkadiuszMarrelli, MariangelaArukha, Ananta PrasadMarshall, JulieArunaksharan, NarayanankuttyMarsico, Antonio DomenicoAsano, KazuhitoMarsili, EnricoAschonitis, VassilisMarsolais, FredericAsekova, SovetgulMarta De Matos, AnaAsghar, Muhammad AhsanMárta, LadányiAsgher, MohdMarta, MonderAsh, DipankarMartellos, StefanoAshapure, AkashMartín Muñoz, AntonioAshraf, Mohammad ArifMartinelli, Adriana PinheiroAshraf, UmairMartínez Ávila, Guillermo Cristian GuadalupeAsif, Hafiz MuhammadMartínez Nicolas, Juan JoséAšperger, DanijelaMartínez Vázquez, Maria LuisaAtanasov, VasilMartinez, CristianAtanassova, MariaMartinez, DaniAthanasiadis, VassilisMartinez, ManuelAthar, Habib-ur-RehmanMartinez, Maria ConcepcionAttila, HegedusMartínez-Antonio, AgustinoAung, May SannMartinez-Garcia, MarinaAussenac, ThierryMartínez-Gómez, PedroAveryanov, Leonid V.Martínez-Hernández, FabiánAvila Sakar, GermánMartínez-Martínez, AlejandroÁvila-Quezada, Graciela D.Martinez-Montero, Marcos EdelAvramidou, EvangeliaMartínez-Moreno, FernandoAxiotis, EvangelosMartínez-Tomé, JuanAyala-Zavala, Jesús FernandoMartín-Gil, JesusAyaz, AsmaMartins, CatiaAycan, MuratMartins, MariaAyeni, AlbertMartins-Loução, Maria AméliaAyora-Talavera, TeresaMartín-Tornero, ElisabetAyoub, IrinyMartynova, ElenaAyoub, Nahla A.Marx, Ítala M. G.Ayyanath, Murali MohanMas, FloreAzad, Obyedul KalamMaschke, RüdigerAzadi, HosseinMaslennikova, Dilara R.Azai, ChihiroMaslov Bandić, LunaAzam, MuhammadMaslov, MikhailAzarin, Kirill VitalievichMasood, AsimAzeem, MuhammadMassimiliano, LauriaAzinheira, Helena G.Masson, Patrick H.Azizinia, ShivaMastalerczuk, GrażynaAzuma, MiraiMasters, EliotAzzini, ElenaMastore, MaristellaBaas, PieterMastrangelo, Anna M.Baasanmunkh, ShukherdorjMasuda, TatsuruBaba, Abu ImranMasuya, HayatoBaba-Moussa, LamineMasyagina, Oxana V.Bąbelewski, PrzemysławMatanguihan, JanetBabosha, Alexander V.Matei, IoanaBaby, André RolimMatejić, JelenaBaccichet, IrinaMaterska, MałgorzataBach, HoracioMáthé, ÁkosBach, Thomas J.Mather, DianeBąchor, RemigiuszMatheson, JustinBácsi, IstvánMathew, RenyBączek, KatarzynaMathias, OttoBaczek-Kwinta, RenataMatias De Alencara, SeverinoBaczewska-Dąbrowska, Aneta HelenaMatichenkov, VladimirBączkiewicz, AlinaMatilla, Angel J.Badaeva, EkaterinaMatin, AnaBadagliacca, GiuseppeMatin, MohammadBadenes-Pérez, Francisco RubénMatkowski, AdamBader, AmmarMatny, OadiBadiani, MaurizioMatos, ManuelBadr, AbdelfattahMatoša Kočar, MajaBadr, AhmedMatraszek-Gawron, RenataBae, Chang-HyuMats, LiliBae, Jae YoungMatsoukis, AristidisBaert, JoostMatsufuji, HiroshiBaeza, RafaelMatsuura, ShoheiBag, SudeepMatthies, DiethartBaghdadi, AliMattii, Giovan BattistaBagniewska-Zadworna, AgnieszkaMattoo, AutarBagnoli, FrancescaMatttila, TapaniBagy, HadeelMatus, José TomásBai, BingMatušinsky, PavelBailly, ChristianMatveeva, TatianaBajgai, YadunathMauceri, AntonioBajguz, AndrzejMaul, ErikaBakacsy, LászlóMauve, CarolineBakaeva, MargaritaMaver, MauroBakalin, VadimMavrodiev, EvgenyBakare, MoshoodMavromoustakos, ThomasBalabanova, VesselaMaxim, AurelBalaes, TiberiusMay, JaredBalaeş, TiberiusMayo-Prieto, SaraBalaguera-López, Helber EnriqueMazeika, RomasBalanza Perez, VicenteMazhar Iqbal, MuhammadBalawejder, MaciejMazur, MajaBalažová, AndreaMazur, RadosławBaldoni, ElenaMazzafera, PauloBaldrich, PatriciaMazzei, PierluigiBalen, BiljanaMazzola, Eugene P.Baležentienė, LigitaMbarki, SoniaBali, SapinderMcBride, KateBalick, Michael J.McCallum, BrentBallen Taborda, CarolinaMcCallum, JasonBalogh, JeremiásMcGroddy, Megan EBalotf, SadeghMcIntosh, MatthewBaltensperger, David D.Mckay, TinaBaltina, LidiaMechchate, HamzaBaluska, FrantisekMechora, ŠpelaBalzano, AngelaMedic, AljazBan, YusukeMedici, Leonardo OliveiraBana, RamMedina Juarez, Luis AngelBanach, ArturMedina, Francisco JavierBandelj, DunjaMedina, RocioBandurska, HannaMedina-Ramírez, IlianaBandyopadhyay, DebasishMedo, JurajBandyopadhyay, SmarakMedyńska-Juraszek, AgnieszkaBanga, Surinder S.Meena, MukeshBankova, VassyaMeftah Kadmiri, IssamBanožić, MarijaMeftaul Islam, Md.Banskota, Arjun H.Meggio, FrancoBantis, FilipposMeghea, AureliaBao, RuiyangMehltreter, KlausBaptista, Cristina M. S. G.Mehraj, HasanBaptista, RafaelMeimandi, Maryam MozafarianBaranová, BeátaMekapogu, ManjulathaBaranova, EkaterinaMekinić, Ivana GeneralićBaraza, ElenaMekky, Reham HassanBarba-Espin, GregorioMeland, MekjellBarbanti, LorenzoMele, GiacomoBarbaric, MonikaMeletiou-Christou, Maria-SoniaBarbedo, Claudio JoséMelgarejo, Luz MarinaBarberini, SaraMelgarejo, PabloBarberon, MarieMelini, ValentinaBarbinta-Patrascu, Marcela-ElisabetaMellano, Maria GabriellaBarbosa, DanielMelosik, IwonaBarbu, VasilicaMelucci, DoraBarcauskaite, KarolinaMelzig, MatthiasBareka, ‪PepyMendes, AndreaBarić, BoženaMendes, PedroBarichello, JessicaMendes, RafaelBarłóg, PrzemysławMéndez-Alonzo, RodrigoBarna, BalázsMendis, Hajeewaka C.Barnett, Ronald D.Mendlerné Drienyovszki, NóraBaron, GiovannaMendoza-Fernández, AntonioBarone Lumaga, Maria RosariaMenezes, Irwin Rose AlencarBarone, BiagioMeng, XiaoxiBarone, EttoreMenghini, LuigiBarone, RobertoMengist, MollaBarreca, DavideMenna, MarialuisaBarreca, SalvatoreMensitieri, FrancescaBarrett, AnnMenzel, Christopher M.Barriocanal, CarlesMerah, OthmaneBarros, Pedro M.Mercuri, Anna MaríaBarros-Galvão, ThiagoMerecz-Sadowska, AnnaBartha, SándorMerenkova, SvetlanaBartnik, MagdalenaMerle Farinós, Hugo BasilioBartolucci, FabrizioMerotto Jr., AldoBartosova, LenkaMesterhazy, AkosBartosz, GrzegorzMészáros, KláraBartoszak-Adamska, ElżbietaMetslaid, MarekBarzanti, Gian PaoloMeudec, EmmanuelleBarzee, Tyler J.Mévy, Jean-PhilippeBasalirwa, DanielMezzalama, MonicaBashir, AftabMezzetti, BrunoBashir, Muhammad AjmalMi, JianingBashir, SafdarMicale, NicolaBashir, SaqibMiceli, FabianoBasile, AdrianaMichael, Vincent Njung’eBaskin, Carol C.Michail, MichailidisBaslam, MarouaneMichailidis, MichailBasnet, Bhoja RajMichalak, MarcinBassi, DanieleMichałek, SławomirBassoli, AngelaMichalska-Klimczak, BeataBasu, Saikat KumarMichard, ErwanBasu, SupratimMichel, PiotrBatish, Daizy R.Mieog, Jos CorneliusBatsalova, TsvetelinaMierek-Adamska, AgnieszkaBattaglia, ValerioMigliore, MelaniaBaturo-Ciesniewska, AnnaMigliori, Carmela AnnaBaum, ChristelMiguel, Maria Da Graça CostaBavaresco, LuigiMihai, BercaBaxevanos, DimitriosMihai, IonBaykov, AlexanderMihailova, GerganaBazha, Sergey N.Mihailović, MirjanaBazos, IoannisMihaljević, InesBeara, Ivana N.Mihaylova, DashaBebeli, PenelopeMikhailova, Ekaterina O.Becker, ClaudeMiki, DaisukeBedoshvili, Yekaterina D.Mikić, SanjaBeeckman, HansMikiciuk, GrzegorzBeers, EricMikulic-Petkovsek, MajaBegović, LidijaMilašinović-Šeremešić, MarijaBehera, Tusar KantiMildažienė, VidaBehiry, Said I.Milella, LuigiBehnke-Borowczyk, JolantaMiler, NataliaBeier, Marcel PascalMileva, MilkaBeilby, MaryMilić, MirtaBekkaoui, FaouziMilioni, DimitraBela, KrisztinaMiliordos, Dimitrios-EvangelosBelbahri, LassaâdMilito, AlfonsinaBelelli Marchesini, LucaMiljković, DanijelaBeliaev, DenisMillan, TeresaBell, KarenMiller, Brandon D.Bellairs, SeanMiller, GloriaBellaloui, NacerMiller, Robert Neil GerardBelle, CristianoMiller, William B.Bellis, Luigi DeMilosavljević, IvanBello-Bello, JericoMin, Cheol WooBellot, María José GómezMin, KyungwonBělonožníková, KateřinaMinafra, AngelantonioBelov, Andrey A.Minasiewicz, JulitaBelova, OlgirdaMinassi, AlbertoBeltran, JesusMinicka, JuliaBelykh, Elena S.Minissale, PietroBen Kaab, SofieneMinkina, TatianaBenchimol-Reis, LucianaMino, MasanobuBenčić, ĐaniMir, ReyazulBendokas, VidmantasMir, RicardoBeneduzi, AneliseMiranda Fernandes, Rafael DreuxBenelli, CarlaMiranda, CristobalBenìtez, EmilioMiranda-Apodaca, JonBenito, Ernesto P.Mircea, CorneliaBentley, Nolan B.Mirmazloum, ImanBento, FatimaMiron, AncaBenvegnú, Dalila MoterMišan, AleksandraBenvenuti, StefaniaMishev, KirilBera, SayantaMisheva, SvetlanaBercu, MioaraMishra, AvinashBergamini, CarloMishra, AwdheshBergero, RobertaMishra, BharatBergougnoux, VéroniqueMishra, Gyan PrakashBeris, DespoinaMishra, RiteshBerkowitz, GeraldMishra, SandhyaBerlanga, Ángel MéridaMishra, SasmitaBernacchia, GiovanniMiśkiewicz, KarolinaBernacki, MaciejMisra, Amarendra N.Bernal, Freddy AlexanderMisra, Rajesh ChandraBernardo, SaraMiszalski, ZbigniewBernhardt, HeinzMitakou, SofiaBernstein, NiritMithoefer, AxelBerrocal-Lobo, MartaMitić-Ćulafić, DraganaBertaccini, AssuntaMitra, MautusiBertamini, MassimoMitrović, MiroslavaBertelli, DavideMitsuya, ShiroBertomeu, Jesus M.Mitsuyama, SusumuBertsouklis, KonstantinosMiyamoto, KojiBęś, AgnieszkaMiyazawa, ShinichiBesendorfer, VišnjaMladenov, VelimirBessho-Uehara, KanakoMladenovic, KatarinaBetekhtin, AlexanderMladenovic, MilanBethke, Paul C.Mlček, JiriBetti, CamillaMlinarić, SelmaBettoni, Jean CarlosMnif, WissemBezirtzoglou, EugeniaMo, YoungjunBezkorovaynaya, IrinaMoar, WilliamBhadra, SouravMoccia, StefaniaBhandari, ManoharMoczkowska, MałgorzataBhandari, SantoshModi, ArpanBhaskar, RakeshModica, GiuliaBhaskara, Govinal BadigerMoffett, PeterBhatia, DharmendraMohamed, HebaBhattarai, KrishnaMohammed, AliBhowmik, PankajMohanan, PadmanabanBhusal, NarayanMohanty, BijayalaxmiBi, GuihongMoise, Adela RamonaBiagi, MarcoMoitzi, GerhardBialek, WojciechMok, Daniel Kam-WahBianchi, DavideMoldovan, Radu-CristianBianchi, GiuliaMolina Salinas, Gloria MaríaBianco, ArmandodorianoMollica, AdrianoBianco, CarmelinaMolnár, Orsolya EszterBianco, RiccardoMolnárová, MariannaBiancolillo, AlessandraMoltchanova, ElenaBiczak, RobertMomčilov, Milana TrifunovićBidabadi, Siamak ShiraniMomma, NoriakiBiel, WiolettaMónaco, CeciliaBieniek, AnnaMonasterio, Romina PaulaBiernasiuk, AnnaMoneim, DiaaBierza, WojciechMonica, ZuzarteBiesiada, AnitaMonreal, José A.Bigler, LaurentMontagnani, ChiaraBijak, MichałMontalbán, Itziar A.Bijak, SzymonMontalvo González, EfigeniaBijelić, ZoricaMontane, Marie-HeleneBilandžija, NikolaMonteiro, ElianaBilia, Anna RitaMonteiro, FilipaBilska-Kos, AnnaMontemurro, CinziaBilska-Wilkosz, AnnaMontenegro, Juan D.Birhan, Yihenew SimegniewMontesano, DomenicoBirsa, Mihail LucianMontesinos Lopez, Juan CarlosBischoff, Adrian A.Montopoli, MonicaBiselli, ChiaraMontoro, PaolaBishnoi, SantoshMontoya, PabloBishop, ChrisMonzote, LianetBiziuk, Marek K.Moparthi, SwarnalathaBizzo, HumbertoMora, FreddyBjörn, Lars-OlofMoraes, Ana PaulaBlakeslee, JoshuaMorais, Maria CristinaBlanco, AntonioMorales, Ana Maria SimonetBlanco, Carlos A.Morales, DiegoBlanco, VíctorMorales, LauraBlanco-Salas, JoséMorales-González, José AntonioBlando, FedericaMorar, CezarBlanke, MichaelMoraru, LuminitaBlattner, FrankMoraru, Paula IoanaBlazevic, IvicaMoravcikova, JanaBlecharczyk, AndrzejMoravec, TomášBoba, AleksandraMoreira, Daniel CarneiroBobis, OtiliaMoreira, Paula MariaBobulska, LenkaMorello, LauraBoccacci, PaoloMoreno, AdrianBocianowski, JanMoreno, Diego A.Boczkowska, MajaMoreno, Maria AngelesBodale, IlieMoreno-Jimenez, Martha RocíoBoddupalli, Prasanna M.Moreno-Ramón, HéctorBode, RobertMorgounov, AlexeyBodor, PéterMóricz, NorbertBog, ManuelaMoriguchi, TakayaBogard, MatthieuMorikawa, MasaakiBogdanov, Ivan V.Morimoto, MasanoriBogo, AmauriMorishita, ToshikazuBohman, BjörnMorita, ShigetoBöhme, MichaelMornar, AnaBohu, TsingMoro, HitoshiBoieiro, MárioMorreale, GiacomoBojarszczuk, JolantaMorrone, JuanBojilov, DimitarMorse, Douglass H.Bojinov, BojinMoshobane, Moleseng ClaudeBojtor, CsabaMoslemi, AzinBokore, FirdissaMostafa, Ahmed M.BölcskeI, HedvigMostafiz, MunirBoldingh, HelenMostowska, AgnieszkaBoldizsár, ImreMosyakin, SergeiBoldura, Oana-MariaMota Poveda, Juan FranciscoBolfe, Édson LuisMota, Ana HenriquesBolpagni, RossanoMota, Manuel Galvão De Melo E.Boltenkov, Eugeny V.Mouga, TeresaBóna, ÁgnesMoulatlet, Gabriel M.Bonchev, GeorgiMourato, Miguel P.Bonciu, ElenaMravec, JozefBoncompagni, EricMroczek, AgnieszkaBonetta, DarioMuangmai, NarongritBonfil, MercedesMuceniece, RutaBoniecka, JustynaMucha, JoannaBonsignore, Carmelo PeterMuhovski, YordanBonvicini, FrancescaMukhtar, TariqBorawska-Jarmulowicz, BarbaraMuktadir, Md AbdulBorek, SławomirMulas, MaurizioBorges, Andrés AntonioMule, PaoloBorgonovo, GigliolaMulet, Jose M.Borisjuk, NikolaiMulla, Sikandar I.Borman, PhilMuller, EmmanuelleBoronnikova, SvetlanaMüller, FrankBorowska-Wykręt, ‪DorotaMüller, MartinBorowska-Żuchowska, NataliaMüller, UlrichBorrego, Eli J.Mulugeta, TewodrosBorrelli, Grazia MariaMuminjanov, HafizBorsai, OrsolyaMumladze, LevanBorsani, OmarMundy, Dion CharlesBorsch, ThomasMunekata, Paulo EduardoBortolami, MartinaMunir, MuhammadBorucki, WojciechMunir, Tariq M.Börve, JorunnMunns, RanaBorza, TudorMunoz Rojas, JesusBoscaiu, MonicaMuntean, DeliaBoscornea, Aurelian CristianMuntean, SorinBosi, GiovannaMural, RaviBoso, SusanaMurata, JunBotez, ElisabetaMurata, KazuyaBotha-Oberholtzer, Anna-MariaMuratani, MasafumiBotticella, ErmelindaMuresan, AndrutaBotton, AlessandroMureșan, Crina CarmenBotu, MihaiMuresan, Mariana EugeniaBoudouresque, Charles FrançoisMurillo, JesusBoufford, DavidMurphy, DenisBougoure, JeremyMushtaq, MuntazirBoumghar, YacineMushtaq, WaseemBouranis, DimitrisMussury, Rosilda MaraBournaris, ThomasMustafa, AdnanBourquin, Leslie D.Mustafa, AhmedBourret, TylerMustafa, AyeshaBousquet, JeanMuthusamy, MuthusamyBowden, BruceMuthusamy, VigneshBoy, ErickMutiga, SamuelBożena, Szewczyk-TaranekMylona, PhotiniBozinou, EleniMylonas, IoannisBožović, MijatMyrta, ArbenBozsó, ZoltánMyśków, BeataBracchetti, LucaNa, Chae-InBrader, GünterNaamala, JudithBrągoszewski, PiotrNacry, PhilippeBraliou, GeorgiaNadal, MiquelBranca, FerdinandoNadal-Sala, DanielBrandão, Rogelio LopesNadeem, MuhammadBrandt, Kali M.Nadeem, Muhammad AzharBrandyk, AndrzejNadporozhskaya, MarinaBrangule, AgneseNaeem, AsifBrat, PierreNagamitsu, TeruyoshiBratek, ZoltánNagdalian, AndreyBratu, AnaNagel, RaimundBräu, LambertNagella, PraveenBrazaitytė, AušraNagy, IstvanBrazee, Nicholas J.Nahar, KamrunBrazzolotto, XavierNaik, AzzaBrčić Karačonji, IrenaNair, Jerald JamesBreaban Iuliana, GabrielaNair, Prakash M GopalakrishnanBrecker, LotharNajar, BasmaBreiterová, KateřinaNajberek, KamilBren, UrbanNajdek Dragić, MirjanaBrennan, Adrian C.Nakamichi, NorihitoBrennensthul, MarekNakamura, ChiharuBreś, WłodzimierzNakamura, HidemitsuBrestic, MarianNakanishi, IkuoBrewer, GaryNakatsuka, AkiraBriedé, JaccoNalewajko, Joanna SekulskaBrighetti, Maria AntoniaNambidiyattil Govindan, ByjuBrígido, ClarisseNanasato, YoshihikoBrkić, AndrijaNandasiri, RuchiraBrogi, Simona RetellettiNandyMazumdar, MonaliBrombin, ValentinaNankar, AmolBrouard, IgnacioNanni, MarcosBrown Miyara, SigalNanos, George D.Broz̃ek, JolantaNapier, RichardBrözel, VolkerNapoleão, Thiago HenriqueBrück, HolgerNardi, SerenellaBrucoli, FedericoNarmani, AbolfazlBrullo, ChiaraNascimento De Queiroz, Sonia ClaudiaBrullo, SalvatoreNascimiento, Luana Beatriz Dos SantosBrumelis, GuntisNash, RobertBrunetti, GennaroNasir Mousavi, Seyed MohammadBrunetti, LeonardoNasir, AbdulBrunetti, LuigiNasiri, AminBrunialti, GiorgioNasiri, JaberBruno, Giovanni L.Nastić, NatašaBruno, MaurizioNatalini, AlessandroBrunori, ElenaNatarajan, PurushothamanBrygadyrenko, Viktor V.Navarra, MicheleBrzosko, EmiliaNavarro-León, EloyBubici, GiovanniNavarro-Pedreño, JoseBubola, MarijanNavazio, LorellaBucher, EtienneNawaz, AhmadBuck, WilliamNawaz, MuhammadBudahn, HolgerNawrot-Chorabik, KatarzynaBudiene, JurgaNayak, DigantBudnikov, DmitryNeag, EmiliaBudryn, GrażynaNebeská, DianaBudzynska, SylwiaŅečajeva, JevgenijaBueno, MilagrosNecas, TomasBujarski, Józef J.Nechita, ConstantinBujdoso, GezaNedělník, JanBulaev, AleksandrNedialkov, Paraskev T.Buldrini, FabrizioNediani, ChiaraBulgakov, VictorNedić Tiban, NelaBulgari, RobertaNeffe-Skocińska, KatarzynaBulychev, Alexander A.Negi, VishalBunce, JamesNegm, AbdelazimBunea, AndreaNegm, WalaaBunea, Claudiu IoanNegrão, SóniaBungau, SimonaNegreanu-Pirjol, TicutaBuonanno, MartinaNegro, CarmineBurbulis, NatalijaNejad, Amir Sasan MozaffariBurczyk, JarosławNeli, Vilhelmova-IlievaBurducea, MarianNelson, DavidBurdziński, JacekNelson, LouiseBurlakovs, JurisNelson, Rex T.Burley, HughNemes, DánielBursac Kovacevic, DanijelaNemes, KatalinBursal, ErcanNemeskéri, EszterBursic, VojislavaNerimetla, RajasekharaBusby, Ryan R.Neriya, YutaroBusconi, MatteoNeta Gostin, IrinaBush, DavidNeto, CarlosBussmann, RainerNeto, LuísBustamante, Ramiro O.Neufeld, HowardBustan, AmnonNeunert, GrazynaBustos, MauricioNevárez-Moorillón, Guadalupe VirginiaButenko, Konstantin O.Nge, Francis J.Butiuc-Keul, Anca LiviaNguyen, Chau N.Butnariu, MonicaNicoletti, MarcelloButrón, AnaNicoletti, RosarioButu, AlinaNiculae, MihaelaBykova, NataliaNiculina, Cosmulescu SinaCaboni, EmiliaNiedbała, GniewkoCabrera-Ponce, José LuisNiedziela, AgnieszkaCacciola, FrancescoNiemann, JanettaCacciola, Santa OlgaNiemczak, MichalCáceres, AlejandroNiemczynowicz, AgnieszkaCagliero, Cecilia LuciaNieto, JorgeCahlikova, LucieNigam, DeeptiCai, GiampieroNigam, ManishaCai, HongmeiNika, Erifili P.Cai, LimingNikalje, GaneshCai, ZheNikolakakis, IoannisCakilcioglu, UgurNikolaou, NikolaosCal, MagdalenaNikolic, LjubisaCaldelas, CristinaNikolić, MiroslavCalderini, OrnellaNikoloudakis, NikolaosCalha, Isabel M.Nikolova, GalinaCalina, DanielaNilsen, Erik T.Călinoiu, Lavinia FlorinaNin, StefaniaCalleja Alarcón, Juan AntonioNiñoles Rodenes, ReginaCalzada, FernandoNishiguchi, MasamichiCamargo-Ricalde, Sara LucíaNishimura, TakeshiCamele, IppolitoNistor, EleonoraCamerlengo, FrancescoNistor, Oana ViorelaCamerlingo, CarloNitulescu, GeorgianaCampbell, Jacqueline D.Nitulescu, MihaiCampbell, Rebecca E.Niu, HaoyuCampera, MarcoNiu, JianfengCampilho, AnaNiu, YujieCampion, BrunoNiwano, YoshimiCampisi, PatriziaNizami, Abdul-SattarCampos, Camila Dalben MadeiraNizhnikov, Anton A.Campos, CatarinaNochera, Carmen L.Campos, Francisco A. P.Nocito, Fabio F.Canales, JavierNodari, RubensCandela, HéctorNoguera Artiaga, LuisCanelo, TaraNoisa, ParinyaCanini, AntonellaNoman, MuhammadCanja, CristinaNoor, AfsanaCano Lamadrid, MarinaNoordergraaf, Inge-WillemCantamessa, SimoneNoori, AzamCantini, ClaudioNosalewicz, ArturCantón, Francisco RuizNosenko, TetyanaCantos, ManuelNosov, Alexander V.Cantú-Silva, IsraelNotaguchi, MichitakaCapasso, RaffaeleNoulèkoun, FlorentCaplan, AllanNoumeur, Sara R.Caponio, FrancescoNoutsos, ChristosCappucci, SergioNovák, JiříCapra, Gian FrancoNovak, JohannesCaprari, ClaudioNovák, PetrCara, Irina GabrielaNovero, MaraCarac, GetaNovikov, AndriyCaracciolo, GiuseppinaNovikova, Lubov YuCarbas, BrunaNowak, ArturCarbone, Dora AllegraNowak, BeataCarbonell, María VictoriaNowakowska, JustynaCardillo, ConcettaNozoye, TomokoCardoso, Jean CarlosNtalli, Nikoletta G.Cardoso, Susana M.Ntouskos, ValsamisCaretto, SofiaNuc, PrzemysławCarletti, GiorgiaNunes Da Silva, MartaCarlson, Craig H.Nunes, CeciliaCarlucci, AntoniaNunes, CristianaCarluccio, Anna VittoriaNuno, RodriguesČarni, AndražNunziata, AngelinaCarnicelli, VeronicaNurzyńska-Wierdak, RenataCarpentieri, AndreaNussbaumer, ThomasCarranca, CorinaNuţǎ, Diana CameliaCarrasco, JaimeNuti, MarcoCarrasquinho, IsabelNyine, MosesCarrer, HelaineNykiel, MałgorzataCarrera Fernández, CeferinoO’Connor, José EnriqueCarrillo Murcia, IreneO’Hanlon, RichardCarrió-Seguí, AngelaO’Rourke, JamieCartaxana, PauloOancea, FlorinCartmill, Andrew D.Ochatt, SergioCartmill, Donita L.Oćwieja, MagdalenaCaruso, MarcoOdagiu, AntoniaCaruso, PaolaOelmuller, RalfCarvajal, MicaelaOfford, CatherineCarvalho, AnaOgiso-Tanaka, EriCarvalho, LuísaOgórek, RafałCarvalho, MárciaOgrodowicz, PiotrCasanova, Michelle T.Oh, Sang-HunCasapullo, AgostinoOh, SungchanCasas, José LuisOhlsen, Daniel J.Cásedas, GuillermoOhno, Katie M.Casertano, MarcelloOhta, ShigeoCasey, Philip S.Ohtomo, RyoCassano, ValeriaOhyama, TakujiCastellanos, AlejandroOkamoto, TakashiCastell-Miller, ClaudiaOkińczyc, PiotrCastilho, PaulaOklešťková, JanaCastillo, AnaOkon, SylwiaCastillo, PabloOkorski, AdamCastro-Guerrero, NormaOksana, OksanaCasucci, CristianoOladosu, YusufCatalán, CesarOlah, Neli KingaCataldo, EleonoraOláh, RóbertCatana, RodicaOlaru, Octavian TudorelCatara, AntoninoOlchowik-Grabarek, EwaCatarino, LuísOlech, MartaCavallaro, ValeriaOlego, Miguel ÁngelČavlović, JuraOlejniczak, IzabellaCazacu, AnaOlejniczak, TeresaCeasar, Stanislaus AntonyOlennikov, DaniilCebola, Maria JoãoOlga, GavrilovaCebulak, TomaszOlivares, Barlin O.Ceccanti, CostanzaOliveira, IsabelCeccarelli, MarilenaOliveira, ManuelaCecílio-Filho, Arthur BernardesOliveira, MozanielCedro, AnnaOlivieri, FabrizioCeliński, KonradOliviu, VostinaruCellini, AntonioOllat, NathalieCendrowski, AndrzejOlle, MargitCeotto, EnricoÖllerer, KingaČepulienė, RitaOlson, Bradley J. S. C.Čerenak, AndrejaOlszewska, Monika ACeresini, PauloOmar, Ashraf M.Černý, MartinOmar, Syed HarisCerqueira, FatimaOmelchenko, Denis O.Cerrato, AndreaOndrasek, GabrijelCerrato, RiccardoOndřej, VladanCerri, MartinaOnete, MarilenaCervantes, EmilioOng, Eng ShiCesar Mateo, Flores-OrtizOniszczuk, AnnaCeschin, SimonaOoi-Kock, TehCesevičienė, JurgitaOppenheimer-Shaanan, YaaraČesonienė, LaimaOprea, ElizaCessna, StephenOracz, KrystynaĆetković, GordanaÖrdög, VinceCha, WonsukOrdoudi, StellaChaban, InnaOrfanidou, Chrysoula G.Chagas-Paula, Daniela A.Orfanoudakis, MichailChakrabarti, SubhadeepOrhan, Ilkay ErdoganChakrabarty, SwapanOrlikowska, TeresaChakraborty, NilanjanOrlov, Yuri LvovichChakraborty, SagnikOrnella, LeonardoChalard, PierreOroian, Mircea AdrianChanderbali, AndreOrtega, Sara FrancoChandran, Anil Kumar NaliniOrtenzi, LucianoChandrasekaran, MurugesanOrtiz De Zárate, DavidChang, LiangOrtiz, AntonioChang, Yu-ChiaOrtiz, SergioChao, Louis KuopingOrtiz-Castro, RandyChao, WangOrton, Thomas G.Char, Si NianOrtúñez, EmmaCharles Richard, John LalithOsada, JesúsCharles, KranowOsei-Bonsu, IsaacCharmet, GillesOslovsky, VladimirChatterjee, PoulamiOsnato, MichelaChaturvedi, RaviOsorio, EdisonChatzidimopoulos, MichaelOstaszewska-Bugajska, MonikaChatzistathis, TheocharisOsterne, ViníciusChaudhry, Usman KhalidOstrowska-Ligȩza, EwaChauhan, NagendraOszako, TomaszCha-um, SuriyanOszvald, MariaChaurasia, SavitaOtt, Peter G.Chavan, PrasadOtulak, KatarzynaChaves, BernardoOtulak-Kozieł, KatarzynaChavez, Adela OlivaOudra, BrahimChavez, FermanOuzounidou, GeorgiaChe, ChuntaoOvando, GustavoCheang, Wai SanOverdieck, DieterChebotar, VladimirOvidi, ElisaChedea, Veronica SandaOwczarek, AleksandraChekanov, KonstantinOwens, VanceChen, Bang-YuanOzarowski, MarcinChen, ChangOzcelik, MineChen, Chang-ChangOzeki, YasuhiroChen, Chih-HuiOżga, WojciechChen, Fure-ChyiOzimec, SinišaChen, GengjunOzimek, EwaChen, HuiOzkan, HakanChen, Jaw RongOzlu, EkremChen, JinmingOzols, MatissChen, KetingPacheco, RitaChen, LiangyuPacholczak, AndrzejChen, PengfeiPacholski, AndreasChen, PoanPaci, MaurizioChen, QiangPacifico, DavideChen, TianxiaoPacuta, VladimirChen, TianyuanPaczos-Grzeda, EdytaChen, Tsan-ChiPaderewski, JakubChen, TuoPadilla, Isabel Maria GonzalezChen, XiaoPaduch-Cichał, ElżbietaChen, XinluPagano, CinziaChen, ZhimingPagano, MarioCheng, Chi-LienPagnotta, Mario A.Cheng, JiayangPagter, MajkenCheng, Juei-TangPaino, Eduardo NotivolCheng, XiaofeiPais, Maria SaloméCheng, YanhaoPaiva, Élder A. S.Chertov, OlegPajak, BeataChervenkov, MihailPajoro, AliceChetschik, IrenePal, KunalChetverikov, Philipp E.Pál, MagdaChetverikov, SergeyPaladines-Quezada, Diego FernandoChhapekar, SushilPalá-Paúl, JesúsChhuneja, ParveenPalermo, Anna MariaChiang, Po-NengPalha, Maria Da GracaChiang, Yu-ChungPalkovics, LászlóChidi, Boredi SilasPallag, AnnamáriaChiellini, CarolinaPalma, DanielaChin, Joong HyounPalma-Bautista, CandelarioChingandu, NomatterPampana, SilviaChinnusamy, ViswanathanPan, ChangtianChiou, Chun-TangPan, LeiqingChițimus, Alexandra-DanaPan, YongbaoChiu, ChinganPan, YuChiu, Kai YingPanasieiwcz, KatarzynaChizhevskaya, ElenaPanayotis, TrigasChizzola, RemigiusPanayotov, MomchilChmara, RafałPanda, Rajendra MohanChmielowska-Bąk, JagnaPanderi, IreneChmura, DamianPandey, Abhay K.Cho, Il-HoonPandey, AshutoshCho, Man-HoPandey, DhananjayCho, Tae-JuPandey, Kapil D.Cho, Won KyongPandey, RenuCho, Yong-GuPandino, GaetanoCho, YuehPandiyan, MuthuramalingamChofong, Gilbert NchongbohPanfilova, OlgaChoi, Il-RyongPang, BoChoi, Kyung-SookPang, YunlongChoi, Myung-SukPang, ZhiliChoi, Won IlPanighel, AnnaritaChoi, Young WhanPanitsa, MariaChong, KangPanja, SaumikChoob, Vladimir V.Pańka, DariuszChooi, KarmunPankou, ChrysanthiChorianopoulou, Styliani N.Panneerselvan, LogeshwaranChou, HongliPanno, StefanoChoudhary, RavishPanov, AlexeyChoudhary, ShrutiPantelidis, GeorgiosChoukr-Allah, RedouanePanteris, EmmanuelChoupina, Altino BrancoPaolacci, SimonaChristos, ChatzisavidisPaolo, DarioChu, ChenggenPaolo, ErmacoraChu, FanghuaPapachristodoulou, AnastasiaChu, Luan LuongPapafotiou, MariaChu, YePapageorgiou, SpyridonChu, ZhaohuiPapaianni, MarinaChukeatirote, EkachaiPapareddy, RanjithChung, HsiaohangPapasotiropoulos, VasileiosChunya, LinPapathanasiou, FokionChupakhin, EvgenyPapini, AlessioChwastek, KrzysztofPaponov, Ivan A.Chwil, MirosławaPapp, IstvánCiampaglia, RobertoPappa, AglaiaCiancio, AurelioPappas, ChristosCiarmiello, LoredanaParani, MadasamyCiccoritti, RobertoParchuri, PrasadÇiçek, Serhat SezaiParedes, AdrianCicuzza, DanieleParisi, MarioCieniawska, BeataPark, Bong-JuCiereszko, IwonaPark, Byung BaeČikeš Čulić, VedranaPark, Hyun-WooCincotta, FabrizioPark, Ky YoungCinelli, FabrizioPark, Kyung-MokCiobotaru, Ana-MariaPark, Sang-WookCiou, Jhih-YingPark, So YonCires, EduardoPark, Yong-JinCirmi, SantaParrella, GiuseppeCirvilleri, GabriellaParsaeimehr, AliCitti, CinziaParus, AnnaCiuffo, MarinaParveen, AmnaCizkova, JanaPârvu, MarcelClapa, DoinaParzymies, MarzenaClark, GregoryPasca, ClaudiaCleal, ChristopherPascual, LauraClements, DavidPasechnik, TatyanaClericuzio, MarcoPasek, JarosławCloonan, KevinPashkovskiy, PavelCoba De La Peña, TeodoroPasković, IgorCocan, IleanaPasławska, MartaCocucci, MaurizioPassalacqua, KarlaCodina, GeorgianaPassalacqua, Nicodemo G.Coelho, Marsen Garcia PintoPassera, AlessandroCoelho, Paula S.Pastenes, ClaudioCoelho, ValentimPasternak, Taras P.Cogato, AlessiaPastirčáková, KatarínaCohen, Ana CarmenPasupuleti, Raghavendra RaoCohen, James IsaacPateiro, MirianCohen, YuvalPatel, Anil K.Coldea, Teodora EmiliaPatel, Jai SinghColecchia, Salvatore AntonioPatel, JineshCollado-González, JacintaPatel, SanjayCollins, CassandraPaternostro, FerdinandoCollum, Tamara D.Pathirana, RanjithColuzzi, RosaPatkowska, ElżbietaConceição, IsabelPatra, Jayanta KumarConde, DanielPatykowski, JacekCondello, MariaPaul, MatthewConesa, María R.Paul, RajeshCongcong, LiuPavan, StefanoCongestri, RobertaPavloušek, PavelConnolly, BryanPavlov, AtanasConstant, PhilippePavlov, DanailConstantinou, Costas S.Pavlovic, DanijelaContaldo, NicolettaPaweł’kowicz, Magdalena E.Conti, StefanoPawlik, AnnaContreras, EstefaniaPawłowicz, IzabelaContreras, Rodrigo A.Pawłowska, BarbaraCook, CatherinePaz Ferreiro, JorgeCooke, BarryPazzagli, LuigiaCooper, Carlton R.Peaucelle, AlexisCopetta, AndreaPecio, AlicjaCopolovici, Dana MariaPedata, Paolo A.Copolovici, LucianPeddanna, KothaCör, DarijaPedersen, HenrikCoram, Robert A.Pedraza, RaúlCordea, MirelaPedreiro, SóniaCordoba, ElizabethPedrosa, Sílvia SantosCórdoba, ManuelPeirano, AndreaCordovilla, María del PilarPejić, IvanCornea-Cipcigan, MihaielaPelageev, DmitryCorrado, GiandomenicoPellegrini, MarikaCorrea-Díaz, ArianPeňázová, EliškaCorreia, Carlos M.Pendergast, ThomasCorreia, PaulaPeneva, VladaCorreia, Sandra IsabelPeng, CongyueCortés, Andrés J.Peng, YaoyaoĆosić, TatjanaPentoś, KatarzynaCosme, FernandaPepper, Alan E.Cosmin Mihai, VesaPeralta, GloriaCosoveanu, AndreeaPeralta-Ruiz, YeimmyCossignani, LinaPerazzolli, MicheleCosta, José HélioPereira, Ana LuisaCostinel, DianaPereira, Ana MartaCourot, EricPereira, CláudiaCouso, InmaculadaPereira, JoseCoutinho, TeresaPereira-Dias, LeandroCozma, PetronelaPereira-Lorenzo, SantiagoCozzolino, DanielPerera, WilmerCozzolino, EugenioPerera-Castro, Alicia VictoriaCozzolino, RosariaPeres, DavidCrain, Benjamin J.Perestrelo, RosaCravero, Maria CarlaPérez De Castro, AnaCrecchio, CarminePérez Reyes, CarolinaCremades Ugarte, JavierPerez, Jose LuisCristina, Romeo TheodorPérez, SeverianoCristofaro, MassimoPérez-Fernández, MaríaCroft, LarryPérez-Lamela, ConcepciónCrosatti, CristinaPérez-López, DavidCrudo, MichelePérez-Marcos, MaríaCrupi, PasqualePeréz-Ramírez, Iza F.Cruz, Jorddy NevesPérez-Romero, Luis FelipeCruz, RebecaPerez-Tornero, OlayaCruz-Hernández, AndrésPerkins-Veazie, PenelopeCruz-Sosa, FranciscoPerlikowski, DawidCsajbók, JózsefPeron, GregorioCsambalik, LászlóPerri, SaverioCseke, KláraPerrino, EnricoCséplö, ÁgnesPerry, NigelCsiszár, JolánPeršić, VesnaCucina, MirkoPertile, GiorgiaCuellar, SaraPeruzzi, LorenzoCuevas, JuliánPervaiz, TariqCullis, ChristopherPerveen, AnjumCunha, JorgePerveen, ShaguftaCupic, TihomirPessoa, Maria FernandaCupina, BrankoPetcherski, AntonCurci, MaddalenaPeters, Reuben J.Curiel Gámiz, José AntonioPeterson, BryanCurtin, ShaunPeterson, JuliaCurutiu, CarmenPetijová, LindaCutulle, MatthewPetkova, ZhanaĆwieląg-Piasecka, IrminaPetoumenou, DespoinaCycoń, MariuszPetriccione, MilenaCzajkowski, RobertPetroni, KatiaCzarnecka, BożennaPetropoulos, SpyridonCzarnocki, ZbigniewPetrou, Athinoula L.Czekala, WojciechPetrová, ŠárkaCzemierska, MagdalenaPetrova, SlaveyaCzerwińska, MonikaPetrovici, Anca-RoxanaCzigle, SzilviaPetruccelli, RaffaellaCzislowski, ElizabethPetruczynik, AnnaCzortek, PatrykPetti, CarloalbertoCzosnek, HenrykPettkó-Szandtner, AladárCzubacka, AnnaPezzotti, GiuseppeD’Addabbo, TrifonePfister, BarbaraD’Agostino, NunzioPfündel, Erhard E.D’Antona, NicolaPham, Anh TungD’Aquino, LuigiPiasecka, AnnaD’Onofrio, ClaudioPiątczak, EwelinaDa Costa, Ricardo M. F.Piazza, StefanoDa Silva Linge, CassiaPicchio, RodolfoDa Silva, Anabela BernardesPiccionello, Antonio PalumboDa Silva, Luis Cláudio NascimentoPichler, AnitaDa Silva, Maria Arlene PessoaPiechalak, AnetaDa Trindade, JúlioPiechota, TomaszDababat, AmerPiedras, PedroDabić, DraganaPiegza, MichałDąbrowska, GrażynaPiepho, Hans-PeterDabrowski, PiotrPiernik, AgnieszkaDadakova, EvaPiesik, DariuszDadáková, KateřinaPietr, StanisławDaferera, DimitraPietras, MarcinDaghma, Diaa E.Pietrella, MarcoDagnon, SoleyaPignata, GiuseppeDalal, AhanPilarska, Agnieszka A.DalCorso, GiovanniPilarski, KrzysztofDalle Fratte, MichelePilati, StefaniaDama, MuraliPilla, RaffaeleDamasevicius, RobertasPillai, SnehaDandlen, Susana AnahiPilu, RobertoDang, Hai-shanPinardi, MonicaDang, JunPiñar-Fuentes, Jose CarlosDanova, KalinaPiñero Zapata, Maria CarmenDanowska-Oziewicz, MarzenaPiñero-Dalmau, DanielDaras, GerasimosPinheiro, JoaquinaDarnet, SylvainPinker, InaDarras, AnastasiosPinto Gomes, CarlosDarwish, EssamPinto, AnaDas, AayudhPinto, Diana Andreia TavaresDas, SauravPinto, EugéniaDas, ShouvikPinto, José Eduardo Brasil PereiraDavies, JuliaPinto, LuísDavis, Aaron P.Piotrowicz-Cieślak, Agnieszka I.Davolos, DomenicoPipan, BarbaraDavydenko, KaterynaPipenbaher, NatasaDavydov, DenisPiqueras, AbelDawes, Warrick R.Pirianov, GrishaDawood, Mona F. A.Pirman, TatjanaDe Aguiar, Paula FernandesPiscitelli, LeaDe Bellis, LuigiPiscopo, AmaliaDe Carlo, AnnaPistelli, LauraDe Caroli, MonicaPitura, KarolinaDe Curtis, FilippoPivić, RadmilaDe Feo, VincenzoPiwowarski, Jakub PatrykDe Feudis, MauroPlader, WojciechDe Graaf, Barend H. J.Plastina, PierluigiDe Jonghe, KrisPlata-Rueda, AngelicaDe La Cruz, MarcelinoPlatre, Matthieu PierreDe La Peña, CleliaPlaza, Blanca M.De La Rosa, Laura AlejandraPobiega, KatarzynaDe La Rosa, LuciaPodder, RajibDe La Rosa, RaulPodgorbunskikh, EkaterinaDe Luca, DanielePodio, MaricelDe Luca, FrancescaPodlasińska, JoannaDe Masi, LuigiPodleśna, AnnaDe Melo, Alberto SoaresPodleśny, JanuszDe Micco, VeronicaPodolska, GrazynaDe Mieri, MariaPodwyszynska, MalgorzataDe Miguel, Maria DoloresPogány, MiklósDe Moraes, Francisca PereiraPogorzelec, MagdalenaDe Muniz, Graciela Bolzón InPogrzeba, MartaDe Oliveira, AlbertoPohlmeier, AndreasDe Oliveira, Ivo VazPoiana, MarcoDe Oliveira, Mozaniel SantanaPoiana, Mariana-AtenaDe Padova, PaolaPoinar, GeorgeDe Pascali, MariarosariaPokhrel, LokDe Santis, Michele AndreaPokovai, KlaraDe Tullio, Mario C.Polesny, ZbynekDe Vries, LisannePolevova, Svetlana V.De, SandipPolidori, PaoloDe, SwarnalokPolidoros, AlexiosDeane, David C.Poljak, IgorDebnath, SashiPoljuha, DanijelaDeepa, PonnuvelPolkowska-Kowalczyk, LidiaDegl’innocenti, DonatellaPollari, MaijaDegtjareva, GalinaPollastro, FedericaDeineko, Elena VictorovnaPommerrenig, BenjaminDeKeyser, Edward ShawnPompermayer Machado, LeviDeKoeyer, DavidPonder, AlicjaDel Amor Saavedra, FranciscoPonnu, JathishDel Buono, DanielePoór, PéterDel Gallo, MaddalenaPop, Lecturer Carmen RodicaDel Giudice, RitaPop, Raluca MariaDel Guacchio, EmanuelePopa, Daniela-SavetaDel Moral Torres, FernandoPopa, IonelDelano, John PaulPopek, RobertDeli, JózsefPoponessi, SilviaDelian, ElenaPopova, AnetaDeligios, Paola A.Popović Đorđević, JelenaDelis, CostasPopovic, BorisDell’Anno, FilippoPopović, BrankoDell’olmo, ElianaPopović, MarijanaDella Bartola, MichelePopović, VladanDellacassa, EduardoPoppinga, SimonDello Ioio, RaffaelePoquet, LaureDemeke, TigstPorceddu, MarcoDemurin, YakovPorcelli, FrancescoDeng, ChaoyiPoretsky, EllyDeng, HuaPorokhovinova, Elizaveta A.Deng, JinyangPorro, DuilioDeng, WeiweiPorta, AmaliaDeng, XiangwenPortela, RubénDenisow, BozenaPortillo-Estrada, MiguelDenoeud, FrancePortis, EzioDent, MajaPossell, MalcolmDepaepe, ThomasPotenza, GiovannaDepeint, FlorePoteser, MichaelDerejko, AdrianaPotokina, ElenaDerewiaka, DorotaPotters, GeertDeryabina, Yulia I.Pottier, JulienDeshmukh, RupeshPottosin, IgorDespland, EmmaPoulíčková, AloisieDesta, Kebede TayePoulios, StylianosDettori, Maria AntoniettaPouliot, RoxaneDetyna, JerzyPouran, HamidDevani, Ravi SureshPoveda, JorgeDevecchi, MarcoPower, HuguesDever, JanePozzi, CarloDevkota, Hari PrasadPrabucka, BeataDevlin, PaulPrado, RenatoDevrnja, NinaPrakofjewa, JuliaDewir, Yaser HassanPramanick, BiswajitDey, AbhijitPramanik, DibyajyotiDeyholos, Michael K.Pranaw, KumarDeyneko, IgorPrasad, Kasavajhala V. S. K.Dhaliwal, Harcharan S.Prasad, Thottethodi Subrahmanya KeshavaDhar, NikhileshPrasasty, VivitriDhariwal, RamanPratta, Guillermo RaúlDi Baccio, DanielaPraz, CoralineDi Lorenzo, CherubinoPrażak, RomanDi Mambro, RiccardoPreiner, DarkoDi Marco, GabrielePreiti, GiovanniDi Martino, CatelloPrenner, GerhardDi Martino, LucianoPrerostova, Mgr. SylvaDi Sansebastiano, Gian-PietroPrieto, JoseDi Sotto, AntonellaPrieto, MiguelDia, MahendraPrimi, RiccardoDiaconeasa, ZoritaPrisa, DomenicoDiana Marcela, BuitragoPrkić, AnteDias, Cindy Leonor AlvesProietti, SimonaDias, Elisabete FurtadoProorocu, MarianDias, Maria CelestePrudencio, Ángela S.Díaz García, PamelaPruett, Christin L.Diaz Pendon, Juan AntonioPrzemysław, NucDiaz, GiselaPrzybył, JarosławDiaz, Tabata Victoria RosasPrzybylska-Balcerek, AnnaDiaz-Maroto, IgnacioPrzybysz, ArkadiuszDickinson, NicholasPshenichnikova, TayanaDickinson, SallyPsychoyou, MariaDictor, Marie-ChristinePszczółkowska, AgnieszkaDiederichsen, AxelPtushenko, Vasilii VitalievichDietzgen, RalfPuac, NevenaDilfuza, EgamberdievaPuccinelli, MartinaDillenberger, Markus S.Puchalka, RadoslawDima, OvidiuPuglia, GiuseppeDimitrova, LyudmilaPullaiah, ThammineniDinache, AndraPuntieri, JavierDinca, LucianPurohit, RiturajDinev, TonchoPurushothama, Charith Raj AdkarDing, ChangjunPuschenreiter, MarkusDing, GuangdaPushkar, SvetlanaDing, LeiPuzanskiy, RomanDing, XiaochunPycia, KarolinaDinică, RodicaPym, AdamDinica, Rodica-MihaelaPyszko, PetrDinischiotu, AncaQaderi, MirwaisDiniz, InêsQaswar, MuhammadDinkins, RandyQayyum, MuhammadDionisio, GiuseppeQi, GuanqiuDistefano, GaetanoQi, ZhaomingDitta, AllahQiu, LinDivashuk, MikhailQiu, MinghuaDixit, NaveenQiu, YinlongDixon, MichaelQu, NingDjalali Farahani-Kofoet, RoxanaQuan, Nguyen VanDługosz, MarekQuerol Audí, JordiDłużniewska, JoannaQuesada Pérez, VíctorDo Amaral Junior, Antonio TeixeiraQuesada, Manuel JamilenaDo Nascimento Vieira, LeilaQuinet, MurielDo, YiyinQuinn, Mark T.Dobreva, AnaQuintana, JuliaDobrikova, Anelia G.Quintanilla, Luis GDobritsa, Anna A.Qureshi, Mohammad IrfanDobrowolska, DorotaQureshi, RahmatullahDobson, Heidi E. M.Raal, AinDodson Peterson, Jean CatherineRabanus-Wallace, Mark TimothyDodueva, IrinaRaccuia, Salvatore AntoninoDoi, KazuyukiRace, MarcoDomblides, Elena A.Racovita, RaduDombrovsky, AvivRacuciu, MihaelaDomina, GianniantonioRadan, MilaDomingos, Idalina J.Radchuk, VolodymyrDomingues, Douglas SilvaRadhakrishnan, ThankappanDomka, AgnieszkaRadić, DankaDonadu, MatthewRadić, SandraDonaire, LiviaRadicetti, EmanueleDong, YueshengRadkowski, AdamDonini, MarcelloRadočaj, DorijanDonovan, NeridaRadocz, LaszloDoppler, MariaRadonić, AniDor, EvgeniaRadotic, KsenijaDorbić, BorisRadu, FierascuDore, AntonioRadu, NicoletaDoroftei, MihaiRadulov, IsidoraDoronila, AugustineRadušienė, JolitaDos Santos, Francisco A. R.Rady, MostafaDosoky, NouraRady, Mostafa M.Douches, DavidRagonezi, CarlaDouvris, ChrisRagusa, SalvatoreDrabińska, NataliaRahal, AnuDragicevic, VesnaRahimmalek, MehdiDragoi, MarianRahman, AnisurDragomir-Balanica, Carmelia MarianaRahman, Inayat UrDragonieri, SilvanoRahmonov, OimahmadDragos, DorinRai, AshutoshDrakulic, JassyRai, Avinash ChandraDraper, IsabelRai, NehaDrašar, Pavel B.Rai, RikkyDreger, MariolaRaimondo, Maria LuisaDremencov, EliyahuRaj Puri, RameshDresler, SławomirRajasekharan, Satish KumarDrobnik, JacekRajendran, RajeswaranDrogoudi, PavlinaRajfur, MałgorzataDrori, ElyashivRajoka, Muhammad Shahid RiazDrost, DanielRajput, VishnuDrummond, Frank Francis A.Raju, RiteshDrzazga, AnnaRakocevic, MiroslavaDrzewiecka, KingaRakosy‑Tican, ElenaDu, MinminRakshit, SujayDu, ZhizhiRaldugina, GalinaDuan, JialeiRamachandraiah, KarnaDuan, TingyuRamadan, Mohsen M.Duan, XuewuRamamoorthy, RengasamyDuan, YuanwenRamasamy, ManikandanDuarte, Gustavo TurquetoRameneni, Jana JeevanDuarte, Maria CristinaRamesh, ManikandanDuarte, NoéliaRamesh, Sunita A.Dubois, AnnickRamirez Ojeda, GabrielaDuckett, Jeffrey G.Ramírez, VicenteDuda, Marcel MateiRamos, JavierDudits, DénesRamos, Maria LucreciaDudnikova, TamaraRamos, PawełDufková, HanaRan, JinhuaDufossé, LaurentRana, Ziaul HasanDuggan, Brendan M.Ranabhat, NarDulić, JovanaRandall, Stephen K.Duliu, OctavianRandi, EttoreDulloo, Mohammad EhsanRangel, LorenaÐulović, AzraRanta, OvidiuDumancas, GerardRapacz, MarcinDumitraș, Adelina F.Râpeanu, GabrielaDunić, Jasenka AntunovićRaposo, RosaDunkić, ValerijaRaptis, DimitriosDunn, Michael F.Rascio, AgataDuque, Luis O.Rascon, AgustinDurairaj, KaliannanRashal, IsaakDuralija, BorisRasheva, IlianaDurczak, KarolRašovský, MarekDurel, Charles-EricRaspor, MartinDurgo, KsenijaRatcliffe, JossDurmus, DorukalpRatcliffe, Richard GeorgeDusenge, Mirindi EricRatet, PascalDushenkov, VyacheslavRathinasabapathy, ThirumuruganDuttke, Sascha H.Rathore, SanjayDutu, LigiaRatiu, AndreeaDuval, Benjamin D.Raudonė, LinaDvořáček, JanRavelonandro, MichelDziadas, Mariusz TomaszRaven, John AlbertEbada, SherifRavetto Enri, SimoneEbeed, HebaRavichandran, MirunaliniEben, AstridRavishankar, Kundapura V.Ebert, AndreasRawat, SwatiEbrahim, WeaamRayns, FrancisEbrahimi, AzizRazgonova, MayyaEbrahimi, NeginRazumova, Olga V.Eckerstorfer, MichaelRazzaq, AbdulEdae, Erena A.Razzaque, SamsadEdmé, Serge J.Reboredo, HenriqueEdwards, James M.Rebucci, RaffaellaEgamberdieva, DilfuzaRébufa, CatherineEgipto, RicardoRector, Brian G.Eguiarte, LuisRedden, RobertEisenback, Jonathan D.Reggi, SerenaEisenstein, EdwardRégine, DelourmeEissa, MamdouhRegni, LucaEl Aouad, NoureddineRehman, AbdulEl Ghorab, AhmedRehman, Shafiq UrEl-Aarag, Bishoy Y. A.Rehrig, ErinElameen, AbdelhameedReid, Michael S.El-Ansary, HosamReim, StefanieElansary, Hosam O.Reinhardt, Jakob K.Elbeaino, TouficReis, AnaEl-Beltagi, HossamReis, CarlosEldahshan, Omayma A.Reis, Filipa S.Elena, Popa MonaReis, MárioEl-Esawi, Mohamed A.Reis, PedroEl-Halawany, Ali M.Reis, RicardoElhawat, NevienRejman, JerzyEliades, MarinosRekasi, MarkEliášová, KateřinaRemizowa, Margarita V.Eliopoulos, Panagiotis A.Remmas, NikolaosElkahoui, SalemRen, ShuaiEl-Keblawy, AliRen, YulinEllouz, OualidRenna, LucianaEl-Nagar, Mehrez E.Resentini, FrancescaElnagar, MohamedReuveni, MosheEl-ramady, HassanReveglia, PierluigiEl-Seedi, HeshamRevilla, PedroElshafie, Hazem S.Rewers, MonikaElshamy, Abdelsamed I.Rewicz, AgnieszkaElsherbiny, ElsherbinyRey, LuisElshobary, MostafaReyes, VincentEl-Tarabily, KhaledReza, Md NasimElumeeva, TatianaRezania, ShahabaldinElvanidi, AngelikiRhazi, LarbiElvira, SusanaRhazi, MohammedElzaawely, Abdelnaser A.Ribaudo, GiovanniEmery, SarahRibeiro Da Silva, Antônio JorgeEmran, Talha BinRibeiro Junior, WalterEngelberth, JurgenRibeiro, AntónioEngels, Johannes M. M.Ribeiro, ArturEnroth, JohannesRibeiro, Paulo RiceliErcisli, SezaiRibeiro-Barros, Ana I.Erdős, LászlóRicachenevsky, Felipe KleinEric Dusenge, MirindiRicelli, AlessandraErland, LaurenRich, Patrick J.Ernst, DávidRichards, NgaioErrea, PilarRichardson, DavidErrico, SimonaRichter, AndreasErst, AndreyRico-García, EnriqueEsatbeyoglu, TubaRigano, LuigiEscamilla Silva, Eleazar M.Riggins, Chance W.Escandón-Rivera, SoniaRighetti, LauraEscobar-Niño, AlmudenaRighini, HillaryEshel, AmramRigó, GáborEsmat, AhmedRijsdijk, Kenneth F.Espen, LucaRincón, VíctorEsperon-Rodriguez, ManuelRio, SimonEspinosa-García, Francisco JavierRios, Juan JoséEspírito-Santo, DalilaRios-Velasco, ClaudioEsposito, SergioRisdian, ChandraEs-Safi, Nour EddineRisk, Michael J.Estep, MattRitter, ManuelEsumi, TomoyaRitter, TimEszter, SugárRivadeneyra-Domínguez, EduardoEudes, AymerickRizvi, Syed Arif HussainEugenios, KokkalouRizzi, CorradoEulenstein, FrankRizzo, ParideÉva, CsabaRobaina, Rafael R.Evans, JulianRobakowski, PiotrEvidente, AntonioRobb, JaneEzra, DavidRoberta, Gagliardi AnnaFabbri, AndreaRoberto, Sergio RuffoFabi, João PauloRobins, JosephFabroni, SimonaRobinson, Sally R.Facundo-Joaquin, Marquez-RochaRobledo Quintos, Norma R.Fadriquela, AilynRobles, PedroFaedda, RobertoRoby, Mohamed Hussein HamdyFagerstedt, KurtRoca-Fernández, AnaFaggioli, FrancescoRocha, CéliaFahad, ShahRochani, AnkitFailla, OsvaldoRoche, BenjaminFaisal, MohammadRodamilans, BernardoFaizan, MohammadRodrigues, António S.Fal, KaterynaRodrigues, Fabrício De ÁvilaFalanga Bolognesi, SalvatoreRodrigues, GonçaloFalcioni, RenanRodrigues, Manuel Ângelo RosaFaligowska, AgnieszkaRodrigues, Márcio José De AbreuFalistocco, EgiziaRodrigues, Maria JoãoFall, MamadouRodrigues, TatianeFallacara, Dawn M.Rodríguez Llorente, Ignacio DavidFan, LushengRodríguez Sousa, Antonio AlbertoFanelli, ValentinaRodriguez, Juan PabloFanfarillo, EmanueleRodríguez, PedroFang, DavidRodriguez-Gutierrez, GuillermoFang, ShengRodríguez-Jimenes, GuadalupeFang, Yan MingRodriguez-Leyva, EstebanFanourakis, DimitriosRodriguez-Rajo, Francisco JavierFaraoni, PaolaRodriguez-Rodriguez, JoséFaria, Jorge M. S.Rodríguez-Seijo, AndrésFarid, MujahidRodriquez, ManuelaFarkas, ÁgnesRoeth, DanielFarkas, AndrásRogozina, Elena V.Farkas, Edit É.Roh, ChanghyunFarmaki, TheodoraRohn, SaschaFarooq, ShahidRojek, JoannaFarooq, TahirRojo Baio, Fábio HenriqueFarris, EmmanueleRollinger, JudithFasano, ValerioRomain, Anne ClaudeFascetti, SimonettaRoman, KamilFasoula, VasiliaRomano, ElioFattorini, SimoneRomanyuk, NataliyaFaure, SébastienRomero, Hernán MauricioFavero, AlessandraRomero, IreneFavre-Réguillon, AlainRónavári, AndreaFazio, AlessiaRonga, DomenicoFecka, IzabelaRonse De Craene, Louis P.Fedonyuk, TetyanaRonzan, MarilenaFedorova, ElenaRopek, DariuszFedosov, V. E.Rosa, RobertFedunov, RomanRosati, LeonardoFeduraev, PavelRosca, MihaelaFeledyn-Szewczyk, BeataRoß, ChristinaFelgueiras, HelenaRoss, IanFelipe-Sotelo, MonicaRossello, Josep A.Fellner, MartinRossi, GabriellaFendrihan, SergiuRossi, MarikaFeng, HaoRotondo, FrancescaFeng, JinghuiRoubelakis-Angelakis, Kalliopi A.Feng, LiRoulia, MariaFeng, MuhuaRoumiantseva, Marina LvovnaFeng, YueRouphael, YoussefFeng, ZhikeRousseaux, María CeciliaFermin, GustavoRoussis, IoannisFernandes De Oliveira, AnaRoussos, Peter A.Fernandes, Manuel M.Routray, PratyushFernández Barbero, GerardoRouws, LucFernández Ochoa, ÁlvaroRoy, ArpitaFernández, Conchi SánchezRoy, JulienFernandez, EduardoRoy, SuchismitaFernandez, EloyRoy, Swapan KumarFernández, Francisco J.Roy, SwarupFernández, HelenaRoychowdhury, RajibFernández, José A.Różalska, SylwiaFernández, Juan A.Rozentsvet, Olga A.Fernández, ManuelRožnovský, JaroslavFernández-Aparicio, MonicaRuan, ChengjiangFernández-Calderón, María CoronadaRuan, Jia-LingFernandez-Ondoño, EmiliaRuan, JianyunFerrante, AntonioRubinowska, KatarzynaFerrarezi, RhuanitoRubio, LuisFerrarini, AlessandroRubio-Casal, Alfredo EmilioFerreira, MarceloRucińska-Sobkowiak, RenataFerreiro-González, MartaRudnicki-Velasquez, PawełFett Neto, Arthur GermanoRudnóy, SzabolcsFeulner, MartinRueda, EdgarFeyissa, Aberham HailuRueda-Puente, Edgar OmarFickert, ThomasRuge-Wehling, BrigitteFidler, JustynaRugienius, RytisFierascu, Radu ClaudiuRui, YukuiFigueiredo, Neusa L. L.Ruiz Téllez, TrinidadFiket, ŽeljkaRuiz, AntonietaFíla, JanRuiz-Garcia, Ana BelenFilardo, FionaRuiz-Sanchez, EduardoFilip, AdrianaRumbou, ArtemisFilippi, Carla ValeriaRumpunen, KimmoFilippo, GeunaRungis, DainisFincheira, PaolaRusinek, RobertFinkeldey, ReinerRusso, AlessandraFinkelstein, RuthRusso, DanielaFiore, Maria CarolaRusterholz, Hans-PeterFischer-Fodor, EvaRustia, Dan Jeric ArcegaFitas, JakubRusu, Marius EmilFleming, MargaretRusu, TeodorFlinn, Barry S.Rutkowska, AgnieszkaFlores, CarolinaRutkowska, MagdalenaFoddai, SebastianoRybczyńska-Tkaczyk, KamilaFodor, FerencRychel-Bielska, SandraFodor, JozsefRymuza, KatarzynaFodorpataki, LaszloRyś, MagdalenaFogarasi, MelindaRysiak, AnnaFoggi, BrunoRyšlavá, HelenaFojtová, MiloslavaRyss, AlexanderFokaides, ParisRyu, ChangseonFonseca, FilomenaRyu, Ki HyunFonseca, Maria Esther de NoronhaSaad, MagedFontana, GianfrancoSabater, BartolomeFontanelli, MarcoSabater-Jara, Ana BelénFontanini, DeboraSabella, ErikaFoo, EloiseSaber, Abdullah AntarFootitt, StevenSabouri, HosseinFormisano, CarmenSabovljevic, MarkoFornoni, JuanSaccà, Maria LudovicaForti, LucaSaccon, FrancescoForzato, CristinaSadeghifar, HasanFotiadis, SiderisSadhukhan, AyanFracassetti, MarcoSadot, EinatFrąckowiak, PatrykSadowski, JacekFraguas-Sánchez, Ana IsabelSadura, IwonaFrancati, SantoloSadykova, VeraFranchi, ElisabettaŠafranko, SilvijaFrancik, RenataSaftić Martinović, LaraFrancikowski, JacekSagarra, Gloria MartínezFrancioni, MatteoSager, ManfredFranke, KatrinSah, Saroj KumarFranková, JanaSaha, BedabrataFranková, LenkaSahoo, Dipak KumarFranzoni, GiuliaŞahpaz, SevserFratini, FilippoSahu, Partha PratimFredotović, ŽeljanaSahuquillo, Elvira BalbuenaFreire, M. SoniaSaiano, FilippoFreitas, AndreiaSaidi, AbbasFrelich, LeeSaifutdinov, BulatFreshwater, WilsonSaini, RupinderFriedt, WolfgangSaiz Jimenez, CesareoFriesen, NikolaiSaiz-Fernández, IñigoFrigerio, JessicaSajadian, Seyed AliFriščić, MajaSajeva, MaurizioFrolov, Andrei N.Sajyan, TonyFrontasyeva, MarinaSakamoto, TakuyaFrugis, GiovannaSakata, NanamiFruk, GoranSala, FlorinFrusciante, SarahSalachna, PiotrFu, FuyouSalah, SalimFu, GangSalaj, TereziaFu, QinSalamon, IvanFu, ShulanSalánki, KatalinFu, XiuminSalanţă, Liana ClaudiaFu, YongbiSalas, Joaquín J.Fuente, VicentaSalava, JaroslavFuentes-Davila, GuillermoSaldarelli, PasqualeFuggi, AmodioSaleem, MuhammadFujii, YoshiharuSaleh-e-In, Md. MoshfekusFujii, YukiSalem, NidaFujino, TakeshiSales, EsterFujita, MasayukiSalgueiro, Lígia RibeiroFukunaga, KenjiSalino, AlexandreFukushima, AtsushiSalmeri, Cristina MariaFülöp, IbolyaSalopek-Sondi, BrankaFunar-Timofei, SimonaSalvi, PrafullFunnekotter, BrynSamara, MariaFunnell-Harris, DeannaSamara, PinelopiFurmanczyk, EwaSamarah, NezarFusco, RobertaSamardžić, JelenaFuta, BarbaraSamec, DunjaFyhrqvist, PiaSamfira, IonelGabay, GiladSamigullin, Tahir H.Gaberščik, AlenkaSamtani, JayeshGabriel, RosalinaSamylina, Olga S.Gabriele, MorenaSánchez Peña, SergioGabrielli, PaoloSánchez, Diego HernánGabryś, BeataSánchez-Estrada, AlbertoGahche, JaimeSánchez-Hernández, EvaGahlaut, VijaySánchez-Romero, CarolinaGaid, MariamSánchez-Vicente, InmaculadaGaile, ZintaSandhu, AmandeepGaillochet, ChristopheSandhu, JaspreetGaj, RenataSandhu, KaransherGajc-Wolska, JaninaSaneoka, HirofumiGajdoš Kljusurić, JasenkaSan-Martín, AurelioGajdosik, Martina SrajerSannino, FilomenaGajdošová, AlenaSano, SatoshiGalan, Ana-MariaSanogo, RokiaGalante, Patricia MussaliSantacruz Varela, AmalioGalanty, AgnieszkaSantamaria, OscarGăldean, NicolaeSantayana, Manuel P.Galeano, Vivian Angelica BernalŠantek, Mirela IvančićGalego, LudovinaSantilli, ElenaGalibina, NatalyaSantini, AntonelloGalić, VlatkoSantos, Armanda E.Gallardo, EugéniaSantos, CarlaGallardo, FernandoSantos, CarmenGallego, Juan Carlos AlíasSantos, MilaGallo, Francesca RomanaSantos, PauloGallo, JosefSantovito, GianfrancoGallo, MonicaSantoyo, GustavoGalunska, BistraSantunione, GiuliaGamarra, RobertoŠaponjac, Vesna TumbasGambacorta, NicolaSaqib, SaddamGambino, GiorgioSarafian, ViktoriaGamboa de Buen, AliciaSarateanu, VeronicaGámez, ManuelSarath, GautamGamrasni, DanSargentis, George-FivosGanatsas, PetrosŠaric̈, LjubišaGancarz, MarekSarkar, AbhijitGandla, Madhavi LathaSarkar, PoulamiGanie, Showkat AhmadSarker, AniruddhaGao, GarySasaki, NobumitsuGao, MinSasanelli, NicolaGao, XiangSato, HikaruGao, YonghongSatriani, AntonioGao, ZongmeiSatyal, PrabodhGárate, Agustín OrmaecheaSaud, ShahGarau, MatteoSaurina, JavierGarayev, Elnur E.Savchenko, Tatyana V.Garbary, DavidSaviano, GabriellaGarcia, Axa GonzalezSavic Gajic, IvanaGarcia, DiegoSavic, Ivan M.Garcia, Juan AntonioSavoi, StefaniaGarcia, LorenzoSawant, ShaileshGarcía, Luis V.Sawicka, BarbaraGarcía-Bores, Ana MaríaSawicki, JakubGarcia-Caparros, PedroSawtschuk, JérômeGarcía-Giménez, RosarioSawyer, AnneGarcía-Gómez, Beatriz EsterSayari, MohammadGarcía-González, VíctorScalabrin, SimoneGarcía-Guzmán, MaríaScariolo, FrancescoGarcia-Jacas, NuriaScarpa, Grazia MariaGarcia-Jares, CarmenScarpato, RobertoGarcía-Lor, AndrésSchachterle, Jeffrey K.Garcıa-Mata, CarlosSchäfer, HannoGarcía-Mauriño, SofíaSchaller, JörgGarcía-Plazaola, José IgnacioSchanzer, IvanGarcía-Ruíz, FranciscoScheidegger, ChristophGardiner, DonaldSchillaci, MartinoGarland-Campbell, KimberlySchindlbacher, AndreasGarnatje, TeresaSchinella, Guillermo R.Garncarek, ZbigniewSchinkovitz, AndreasGarrido, CarlosSchinnerl, JohannGarvey, MichaelSchläpfer, JoëlleGasic, UrosSchlossberg, Maxim J.Gasparro, MaricaSchmidt, LilianGassaway, Brandon MarkSchmidt, MathiasGastal, FrançoisSchmidt-Wolf, Ingo Gh H.Gates, ColinSchmitt-Keichinger, CorinneGauch, HughSchnabel, UtaGaudin, ValérieSchneider, AnnaGaudio, LucianoSchneider, Yves-JacquesGautam, SaurabhSchneitz, KayGavazzi, GiuseppeSchoberer, JenniferGavelienė, VirgilijaScholtz, VladimírGavrila, Adina IonutaSchouten, AlexanderGavrilas, SimonaSchreiber, Karl J.Gavrilenko, TatjanaSchröder, VerginicaGavrilova, Olga P.Schroeter-Zakrzewska, AnitaGavrilović, AnaSchubert, IngoGawęda, DorotaSchuehly, WolfgangGaweł, ElizaSchueller, Michael JohnGaweł-Bęben, KatarzynaSchultz, StewartGawłowska, MagdalenaSchulz, AlexanderGawroński, JacekSchulz, MargotGawronski, StanislawSchulze, ChristianGaysina, LiraSchulze, StefanieGazoulis, IoannisSchuster, MirkoGdula-Argasińska, JoannaSchweigkofler, WolfgangGe, XiaoweiŚcibisz, IwonaGeary, Timothy G.Sciubba, FabioGębarowska, ElżbietaScoditti, EgeriaGecheva, GanaScopa, AntonioGediga, KrzysztofScott, IanGeng, QifangScrano, LauraGennaro, MassimoSebbenn, Alexandre MagnoGenovese, ClaudiaSeben, VladimirGenovese, FrancescoSedláková, BoženaGentile, CarlaSedlakova-Kadukova, JanaGentili, DenisSeghal, AkankshaGentili, RodolfoSegura, AnaGeorgakopoulos, Dimitrios G.Seijas Vázquez, Julio A.George, JustinSeitz, BjoernGeorgescu, CeciliaSękara, AgnieszkaGeorgescu, EmilSekutowski, TomaszGerbig, StefanieSelas, VidarGergócs, VeronikaSeleiman, Mahmoud F.Gerke, JörgSelvakesavan, RajendranGerm, MatejaSelvaraj, AnthonymuthuGermano, Maria PaolaSelvaraj, ArokiyarajGerrano, Abe ShegroSelvaraj, ChandraboseGerszberg, AnetaSemagn, KassaGevrenova, RenetaSemeniuc, Cristina AnamariaGhaderiardakani, FatemehSemerdjieva, Ivanka B.Gharby, SaidSemida, WaelGhilardi Lago, João HenriqueSempruch, CezaryGhimire, BalkrishnaSemwal, Deepak K.Ghimire, Bimal KumarSen, Madhab KumarGhogare, RishikeshSenger, ElisaGhorbani, AbazarSennikov, AlexanderGhuge, SandipSensuła, BarbaraGiacomin, RenataSeo, Hak SooGiamperi, LauraSeo, Myung-JiGianinetti, AlbertoSeo, ShigemiGiannoulis, KyriakosSepcic, KristinaGiannoutsou, EleniSequeda-Castañeda, Luis G.Gieczewska, KatarzynaSera, BozenaGietler, MartaSeregin, Ilya VladimirovichGifford, MiriamSergeev, MichaelGilca, MarilenaSergio, LucreziaGil-Pelegrín, EustaquioSerkan Selli, SerkanGiordani, PaoloSerpe, MarceloGiordano, CristianaSerrano, Dolores R.Giordano, MariaSerrano, MaríaGiordano, SimonettaSerrano, RitaGiorgi, VeronicaSestras, Adriana F.Giovanneti, ManuelaSestras, RaduGiovino, AntonioSeth, Chandra ShekharGîrd, Cerasela ElenaSetter, Tim L.Girich, ElenaSetzer, William N.Giroux, MikeSevastre, BogdanGitsopoulos, ThomasSevcovicova, AndreaGiuffrida, SalvatoreSeverns, Paul M.Giulia, MattaliaSevilla-Morán, BeatrizGiunti, GiuliaSferrazza, GianlucaGiurg, MirosławSganzerla, WilliamGiussani, LilianaSgaragli, GiampietroGladish, Daniel K.Sghaier-Hammami, BesmaGładyszewska, BożenaShaaf, SalarGlasa, MiroslavShafiei, RezaGleń-Karolczyk, KatarzynaShafiq, SarfrazGligor, Felicia GabrielaShah Jahan, MohammadGlina, BartłomiejShah, KamalGnanasekaran, PrabuShahid, Muhammad FaheemGnanesh, Belaghihalli N.Shahida, BushraGniazdowska, AgnieszkaShahinnia, FahimehGo, RuseaShahzad, AliGobin, IvanaShahzad, AnwarGodlewska, PatrycjaShakeel, MuhammadGoggin, Danica E.Shakoor, AwaisGohari, GholamrezaShamshiri, Redmond R.Gokul, ArunShan, WeiGołąbek-Sulejczak, DorotaShanab, Sanaa Mahmoud MetwallyGolan, KatarzynaShannon, LauraGolani, LalitShapiro, ArthurGolczyk, HieronimSharafutdinov, Irshad S.Goldenkova-Pavlova, IrinaSharma, AjayGoldstein, RinaSharma, KavitaGoldwasser, YaakovSharma, Mahaveer P.Gołembiowska, KrystynaSharma, MinaxiGolia, Evaggelia E.Sharma, Pradeep KumarGolinelli, Marie-PierreSharma, SunnyGolob, AleksandraSharma, TriptiGolokhvast, KirillSharma, VeerendraGolubkina, NadezhdaSharma, VikasGomes, FernandaSharoni, YoavGómez López, PedroSharpe, ShaunGómez Minguet, EugenioShchennikova, AnnaGomez Roldan, Maria VictoriaShekhawat, Narpat S.Gómez, FernandoShelake, Rahul MahadevGomez, KarenShen, ChaoGómez-Cruz, IreneShen, JianlinGómez-Martín, CristinaShen, XiangjinGomez-Merino, FernandoSheng, HuajinGómez-Ramírez, AnaSheng, OuGomis-Cebolla, JoaquinSheng, RuilongGomoiu, IoanaSheppard, PaulGömöry, DušanSherstneva, OksanaGomoryova, ErikaSheshnitsan, SergeiGonçalves, Ana C.Sheteiwy, MohamedGonçalves, Maria Sameiro T.Sheval, EugeneGonçalves, Micael F. M.Shi, RuiGonçalves, SandraShi, ZhaoyongGoncalves-Esteves, VaniaShibaeva, Tatjana G.Goncharova, Olga. Yu.Shikov, Anton E.Gonda, SándorShilev, StefanGong, DiShimabukuro, YosioGong, HaijunShimizu, KazuoGontcharov, Andrey A.Shimizu-Inatsugi, RieGontier, EricShinde, HarshrajGonzález Laredo, Rubén F.Shinomura, TomokoGonzález, AlbertoShishova, Maria F.González, José A.Shkondrov, AleksandarGonzález, Juan M.Shneyer, VictoriaGonzález, María CruzShojaei, SaeedGonzalez, MauricioShokat, SajidGonzález, RubénShrestha, JibanGonzalez-Benito, Maria ElenaShtein, IlanaGonzález-Cortazar, ManasésShu, ChanglongGonzález-De-Peredo, Ana V.Shuai, XiufuGonzález-Domínguez, ElisaShuh Ichi, NishikawaGonzález-Hernández, Ana I.Shukherdorj, BaasanmunkhGonzález-López, ÓscarShukla, MukundGonzález-Minero, Francisco JoséShukry, MustafaGonzález-Trujano, Ma. EvaShults, Elvira E.Gonzalo, SorianoShumilina, Daria. V.Gorny, AdrienneShuyskaya, ElenaGorpenchenko, Tatiana Y.Siadjeu, ChristianGorshkov, VladimirŠic Žlabur, JanaGórska, Ewa BeataSiddique, Muhammad IrfanGórska-Drabik, EdytaSiddiqui, Manzer H.Goryunova, SvetlanaSieczko, LeszekGospodarek, JaninaSiejak, PrzemyslawGosztola, BeátaSiemianowski, OskarGoto, ShingoSieniawska, ElwiraGotor, Alicia AyerdiSienkiewicz, StanislawGoto-Yamada, ShinoSieprawska, ApoloniaGötz, Klaus-PeterSierocka, MałgorzataGoud, Burragoni SravanthiSigillo, LoredanaGoulart Fontes, José CarlosSilchenko, Alexandra S.Goutam, UmeshŠiler, BranislavGovindhan, GanesanŠilha, DavidGozdowska, MagdalenaSilkova, Olga GennadievnaGozmanova, MariyanaSillero, NeftalíGrabarczyk, MałgorzataSilva, Amelia M.Grabau, Larry J.Silva, AuroraGrabowski, PaulSilva, EnilsonGradstein, Stephan RobbertSilva, GoncaloGrafi, GideonSilva, Helena GomesGrahovac, NadaSilva, Manuela Moreira DaGramig, GretaSilva, Marcos José DaGranica, SebastianSilva, Maria HelenaGrassi, FabrizioSilva-Espinoza, Brenda A.Grațiela Vicaş, LauraSIlva-Rosales, LauraGrativol, CliciaSilveira, DâmarisGrauda, DaceSilver, MichaGrdiša, MartinaSilvestre, SamuelGreer, DennisSilvestri, CristianGregor, ThomasSilwal, AbhiseshGreisen, Per Jr.Sima, Nicusor-FlaviusGrewal, JasneetSima, Rodica MariaGrichar, W. JamesSimard, Marie-JoséeGriffin, KevinSimeonov, VasilGriffiths, JonathanSimirgiotis, MarioGriga, MiroslavSimkin, Andrew J.Grigore, Marius-NicusorSimm, StefanGrinzato, AlessandroSimmons, Carl R.Grishko, VictoriaSimon, Maria RosaGrizanova, Ekaterina V.Simon, TaugourdeauGrljušić, SonjaSimona, Strgulc KrajšekGroninger, JohnSimone, NicolaGrosso, ClaraSims, JamesGrover, AnilSinapidou, EvangeliaGruber, James V.Singab, Abdel-Naser B.Grubešić, Renata JurišićSingh, AbhijeetGruda, Habil. N.Singh, AvtarGruhlke, Martin C. H.Singh, B. K.Grulova, DanielaSingh, BhupinderGrumet, RebeccaSingh, Bir BahadurGrusz, Amanda L.Singh, HarbinderGrygansyi, AndriiSingh, HarminderGrzebelus, DariuszSingh, KashmirGrzebelus, EwaSingh, ParbeenGrzebisz, WitoldSingh, PhougeishangbamGrzędzicka, EmiliaSingh, RakeshGrzegorczyk, IzabelaSingh, Rupesh KumarGrzelak-Błaszczyk, KatarzynaSingh, Shiv VendraGrzybowski, MarcinSingh, Suraj KumarGu, GanyuSinghania, Reeta RaniGuan, XinSinha, Rajeev R.Guarino, CarmineSinha, RanjitaGudz, NataliaSinjushin, AndreyGudžinskas, ZigmantasSipman, Harrie J. M.Guerineau, FrançoisSipos, GyörgyGuerra-Sanchez, GuadalupeSipos, LászlóGuerrero, José Alejandro HerediaSipos, PéterGuerrero, YolandaSîrbu, Carmen EugeniaGuerrero-Sanchez, Victor M.Sisto, FrancescaGuetat, ArbiSitarek, PrzemyslawGuevara Garcia, Angel ArturoSitko, KrzysztofGuevara, María GabrielaSiudem, PawełGuevara, RamónŠiukšta, RaimondasGugliuzza, GiovanniSivanesan, IyyakkannuGuha, SujaySiwach, PriyankaGuil-Guerrero, José LuisSiwik-Ziomek, AnettaGuillén-Bejarano, RafaelSkalický, MilanGuillén-Sánchez, Dominico A.Skalska-Kamińska, AgnieszkaGuillotin, BrunoSkenderidis, ProdromosGullì, MariolinaSkoczylas, ŁukaszGultyaeva, ElenaSkorupa, MonikaGünthardt-Goerg, Madeleine S.Skot, LeifGuo, BinSkoufogianni, ElpinikiGuo, FenghaiSkowrońska, MonikaGuo, LiangSkrt, MihaelaGuo, Ming-QuanSkrypnik, Liubov N.Guo, XiaoSkrzypek, EdytaGuo, YiqingSkuhrovec, JiriGuo, ZhanyongSkutnik, EwaGupta, AartiSkwierawska, AnnaGupta, Adarsh K.Sladonja, BarbaraGupta, RaviSlater, DuncanGupta, Sanjay K.Ślesak, HalinaGururani, Mayank AnandSlim, SmaouiGurusinghe, SaliyaSlimestad, RuneGurvich, Diego E.Śliwińska-Wilczewska, SylwiaGusella, GiorgioSłomka, AnetaGutekunst, KirstinSlomnicka, RenataGutensohn, MichaelSlovák, MarekGutierrez Gamboa, GastonSlováková, ĽudmilaGutierrez Martinez, PorfirioSłupski, JacekGutiérrez, SalvadorŚlusarczyk, SylwesterGuzmán, José MiguelSmalle, Jan A.Gyamera, Ebenezer AntwiSmaranda, CameliaGyöngyvér, MaraSmedbol, ÉliseHa, GeonsooSmercina, DarianHaack, BarrySmeriglio, AntonellaHabuš Jerčić, IvankaSmeti, EvangeliaHack, EthanSmith, ElishevaHackney, BelindaSmolander, Olli-PekkaHadidi, MiladSmoleń, SylwesterHaeng-Hoon, KimSmolikova, GalinaHafez, Elsayed E.Smolinska, BeataHafez, EmadSmykal, PetrHagidimitriou, MariannaSneideris, DonatasHaigh, AnthonySnoussi, MejdiHakeem, Khalid RehmanSoares, CristinaHakme, ElenaSoares, Pedro R.Halbert, Susan E.Sobeh, MansourHalitschke, RaykoSobiech, ŁukaszHalley, JohnSoceanu, AlinaHallmann, EwelinaSocha, RobertHam, Tae-HoSochacki, DariuszHamamoto, ShinSöderström, LarsHamberger, BjörnSoengas, PilarHambuckers, AlainSofiene, Ben KaabHameed, AmjadSofy, MahmoudHameed, KhalidSoga, KouichiHamerlynck, Erik P.Sogawa, NorioHamman, JoiasSogorb Sánchez, Miguel ÁngelHammerschmidt, RaySohrab, Sayed SartajHamza, AlaaEldin A.Soica, CodrutaHamzelou, SaraSokolov, DenisHan, FangpuŠola, IvanaHan, GuominSolberg, SveinHan, Kyu YeonSolhaug, Knut AsbjørnHan, YongSoliman, MohamedHan, YuyanSolomina, Olga N.Hanaka, AgnieszkaSolovchenko, AlexeiHanba, Yuko T.Soloviev, AlexanderHancevic, KatarinaSolovyev, AndreyHancock, Charles NathanSoltabayeva, AigerimHancu, GabrielSomeya, NobutakaHangan, AdrianaSommaggio, DanieleHanganu, DanielaSomu, PrathapHannoufa, AbdelaliSon, Chang-HwanHano, ChristopheSon, Ki-HoHanus, PawełSon, Young-OkHanus-Fajerska, Ewa JoannaSonah, HumiraHao, YueSong, Jun-HoHaralampidis, KosmasSong, Kwan-JeongHarasim, ElżbietaSong, Yang-HeonHardies, Stephen C.Sonobe, ReiHarman, GarySopeña-Torres, SaraHarris, DavidSorgonà, AgostinoHârța, MonicaSoria Garcia, Juan MiguelHartmann, AntonSorokan, AntoninaHartung, JensSorokina, Irina. V.Harun, SarahaniSorrentino, AngelaHasan, MahadiSorrentino, Maria CristinaHasan, Syed AimanSorrentino, RobertoHasanaliyeva, GultakinSorribas, Francisco JavierHasanuzzaman, MirzaSosa Díaz, TeresaHase, YoshihiroSosulski, TomaszHasegawa, MorifumiSoto, ÁlvaroHaselton, Aaron T.Soto-Cerda, Braulio J.Hashem, AmrSottile, FrancescoHashem, MohamedSõukand, RenataHashida, KohSousa, Joana L. C.Hasiów-Jaroszewska, BeataSousa, Maria JoãoHassan, ErrolSousa, Rose MarieHassan, MahmudulSouza, Gustavo MaiaHassan, MohammedSouza-Chies, Tatiana TeixeiraHassan, Saad El-DinSowa, IreneuszHassandarvish, PouyaSowa, SylwiaHassanpouraghdam, Mohammad BagherSowinski, JozefHassel, KristianSowndhararajan, KandhasamyHatakeyama, KatsunoriŠpanič, ValentinaHausladen, HansSpanò, CarmelinaHavaux, MichelSpasov, Alexander A.Havel, LadislavSpetz, Carl Jonas JorgeHavlin, John L.Spiegler, VerenaHawrylak-Nowak, BarbaraSpissu, YleniaHayat, ShamsulSportelli, MinoHayes, FelicitySprio, Andrea ElioHayward, AliceSrečec, SinišaHazai, LászlóSreedasyam, AvinashHazubska-Przybył, TeresaSreenivasan, SasidharanHe, BaoyeSreerama, LakshmaiahHe, GuangyuanSridhar, KandiHe, LiangliangSrinuanpan, SirasitHe, WeimingSrivastava, Anoop KumarHe, XuexiangSrivastava, Dhruv AdityaHe, YuqingSrivastava, RenuHebbar, Kukkehalli BalachandraSroka, ZbigniewHedden, PeterSrur, Ana M.Hefferon, KathleenStachiv, IvoHegazy, Mohamed-Elamir F.Stachula, PaulinaHegedűs, AttilaStachurska-Swakoń, AlinaHegedusova, AlzbetaStahl, Randal S.Heinbockel, ThomasŠtajner, NatašaHeinrich, FelixStalažs, ArtursHeinrichs, SteffiStan, LauraHejátko, JanStaniak, MariolaHelms, AnjelStanislaus Antony, Antony CeasarHemkemeyer, MichaelStankiewicz-Kosyl, MartaHenao-Rojas, Juan CamiloStanković, JelenaHenriques, JoanaStarowicz, MałgorzataHenry, BrienStarzak, KarolinaHernández, CarmenStaszak, Aleksandra MariaHernández, FranciscaStaszek, PawelHernández, Luis E.Stavolone, LiviaHernández, VíctorStavridou, EvangeliaHernández-Fuentes, Alma DeliaStazi, Silvia RitaHernández-Prieto, MiguelStebel, AdamHernández-Ruíz, JosefaStefan, Daniela SiminaHerrera, HectorŠtefan, Maja BivalHerrero, BaudilioStefan, SimmHesami, MohsenŠtefančík, IgorHesse, Benjamin D.Stefanescu, RuxandraHessini, KamelŠtefanić, EditaHetherington, Alexander J.Stefaniu, AmaliaHeulin, ThierryStefanovic, OlgicaHewedy, OmarStefanowicz-Hajduk, JustynaHidalgo Vidal, AlyssaStefanucci, AzzurraHigley, LeonStefanut, SorinHijazi, AkramSteinmacher, Douglas A.Hilario, ElenaSteliga, TeresaHildebrand, DavidStepanova, AnnaHillyer, JulianStephenson, ChristianHiltunen, LeaStępień, ArkadiuszHinojosa, LeonardoSteponavičienė, VaidaHirara, ToshiyukiSteuer, ChristianHirota, MitsuruStevenson, DennisHisatomi, OsamuStewart, CharlesHnilicka, FrantisekStirbet, AlexandrinaHnilickova, HelenaStoian, VladHo, Tsing-FenStojanovic, NikolaHo, Yi-ChengStojceska, ValentinaHocart, CharlesStojković, DejanHochwagen, AndreasŠtolfa, IvnaHodge, SimonStorchi, PaoloHofbauer, WolfgangStorer, NicholasHoffman, AryStoyanova, AlbenaHöglind, MatsStoyneva, MayaHolalu, Srinidhi V.Stpiczyńska, MałgorzataHolb, ImreStrati, Irini F.Holt, Scott M.Středa, TomášHolton, TimothyStričević, RužicaHolub, PetrStroheker, SophieHolubec, VojtechStrub, Daniel JanHong, Chi-eunStrube, JochenHong, Jong ChanStrzemski, MaciejHong, YanStudnicki, MarcinHoon Lee, JeongStuessy, TodHooykaas, PaulStushnoff, CecilHoque, Md. AnamulStütze, ThomasHorchani, HabibStützel, ThomasHordyjewicz-Baran, ZofiaSu, HuananHorel, ÁgotaSuassuna, Nelson DiasHori, KiyosumiSubbotin, Sergei A.Horst, Egon HenriqueSubramani Paranthaman, BalasubramaniHorstman, AnnekeSubramanian, ParthibanHorvat, DanielaSubramanian, SowmyalakshmiHorvatek, DanijelaSubramanyam, RajagopalHorváth, EditSuchowilska, ElżbietaHosokawa, YoshitakaSugai, TetsutoHoson, TakayukiSugier, PiotrHossain, Md AmzadSugimoto, KoichiHossain, Mohammad BelalȘuică-Bunghez, RalucaHotos, George N.Sujak, AgnieszkaHou, HongweiSujkowska-Rybkowska, MarzenaHoung, Jer-YiingSukhodolskaya, RaisaHouse, MeganSukhorukov, Alexander P.Howell, PhilSukhova, EkaterinaHoyerova, KlaraSuleymanov, RuslanHrckova, GabrielaSułkowska, MałgorzataHrevušová, ZuzanaSułowicz, SławomirHric, PeterSumenjak, Tadeja KranerHrivnák, MatúšSun, ChangHsia, Shih-MinSun, FengjieHsieh, ChangweiSun, ShaolongHsieh, Lu-ShengSun, YaliHsieh, Ming-JuSung, Tae-EungHsieh, Wen-TsongSung, Wen-ChiehHsu, Chuan-ChihSunny, TomHsu, Ssu-WeiŠupljika, FilipHsu, StephenSuprun, Andrey R.Hsu, Yu-ChiaSur, IoanaHsueh, Yuan-ShuoSuriharn, BhalangHu, ChenlinSurina, BoštjanHu, GongsheSurówka, EwaHu, HaixiaoSusanna, AlfonsoHu, JieSushkova, SvetlanaHu, KainingSusila, HendryHu, LiyongSut, StefaniaHu, XiaohuiSutan, AncaHu, XiaopingSuzui, NobuoHu, ZanminSuzuki, TakahiroHua, JianfengSuzuki, YoshihitoHua, KhuongSvanberg, IngvarHua, YingpengŠvedienė, JurgitaHuang, Chien-JuiSvetec Miklenić, MarinaHuang, Chien-YuŠvubová, RenátaHuang, FuchenSwarup, SanjayHuang, Haw-MingSwiecilo, AgataHuang, Hui-ChiŚwierczyński, SławomirHuang, JianliangSwift, JosephHuang, JianzhiSwify, SamarHuang, LiliŚwit, PawełHuang, LinkaiSwoczyna, TatianaHuang, PeichengSykacek, PeterHuang, YanboSykłowska-Baranek, KatarzynaHuang, YibinSymanowicz, BarbaraHuang, YunlongSymonds, RachaelHubbard, MichelleSynowiec, AgnieszkaHuchzermeyer, BernhardSyrpas, MichailHugouvieux, VéroniqueSytar, OksanaHuh, Sung UnSytykiewicz, HubertHusen, AzamalSzabo, AndrasHussain, Abdullah I.Szabo, MilanHussain, AmjadSzabó, SándorHussain, AnwarSzakiel, AnnaHussain, BabarSzalay, LaszloHussain, HidayatSzara, EwaHussain, MubsharSzczepanek, MałgorzataHussain, SaddamSzczepaniak, OskarHutorowicz, AndrzejSzczuka, EwaHutu, IoanSzepesi, AgnesHwang, Dong-jinSzewczyk, AgnieszkaHyskova, VeronikaSzewczyk, KatarzynaIannacone, RinaSzmidt, Alfred EdwardIasur-Kruh, LilachSzpyrka, EwaIbrahim, IskanderSzűcs, ZsoltIbrahim, SabrinSzulc, PiotrIchimura, KazuoSzumny, AntoniIchinose, KatsuyaSzwengiel, ArturIchitani, KatsuyukiSzymandera-Buszka, KrystynaIelciu, IrinaSzymanowska-Pułka, JoannaIevinsh, GedertsSzymańska, GrażynaIgnatov, AlexanderSzymańska, MagdalenaIio, AtsuhiroSzymański, MarcinIjaz, MuhammadSzymczyk, PiotrIkeda, Tatsuya M.Tabanca, NurhayatIkeda, YoshihisaTabara, MidoriIkehara, KenjiTada, YuichiIkkonen, Elena N.Tadić, VanjaIkoyi, IsraelTaglienti, AnnaIlek, AnnaTaguchi, KazunoriIlić, JelenaTaher, MuhammadIliev, MarioTahir, AmmarIlieva, Yana E.Takahashi, ShinyaIllés, ÁrpádTakahashi, ToshiyukiIlyas, MuhammadTakahiro, YonezawaImbimbo, PaolaTakakura, TadashiImran, MuhammadTakano, AtsukoImran, ShahinTakashi, IshidaIndreica, AdrianTakayama, SeijiIngelbrecht, IvanTakayanagi, MasaoInnangi, MicheleTakeshima, RyomaInomoto, Mário MassayukiTakhter, Ramandeep S.Intrigliolo, Diego S.Takita, MarcoIordachescu, MihaelaTallarita, AlessioIorizzi, MariaTamagnone, MarioIovene, MarinaTamasi, GabriellaIovieno, PaoloTamburino, RacheleIqbal, AmjadTan, ZhiyuanIqbal, JavedTanaka, TakashiIqbal, MuhammadTanase, CorneliuIqbal, NasirTanco, SebastianIqbal, ShahidTang, AlvinIqdiam, BasheerTang, RenjieIrfan, MohammadTang, XiangruIrmina, Maciejewska-RutkowskaTang, YuIrving, LouisTangherlini, Timothy R.Isayenkov, StanislavTanneberger, FranziskaIseppi, RamonaTanveer, MohsinIsermann, MaikeTao, XuanyuIsfahani, Mehdi NasrTarakanov, IvanIshikawa, ShokoTarbeeva, DaryaIshizawa, HidehiroTardella, Federico MariaIslam, Md TariqulTardugno, RobertaIslam, Md. RafiqulTareq, Fakir ShahidullahIslam, Mohammad ZahirulTarkowski, PetrIslam, MonirulTarola, Anna MariaIslam, RabiulTarquini, GiuliaIslam, TofazzalTarraf, WaedIslebe, Gerald AlexanderTarricone, LuigiIsmaiel, AdnanTartaglia, AngelaIsmail, MohannadTartaglia, MariaIsman, Murray B.Tassi, LorenzoIsola, DanielaTataridas, AlexandrosIstace, BenjaminTava, AldoItabashi, YutakaTavladoraki, ParaskeviIto, HisashiTayade, RupeshIto, ShinsakuTchetina, ElenaIturriaga, GabrielTchórzewska, DorotaIturritxa, EugeniaTeacǎ, Carmen AliceIvanov, AlexanderTeaumroong, NeungIvanov, Peter A.Tedeschi, AnnaIvanova, DanielaTedesco, IdoloIvanova, LarissaTeh, Soon LiIvanova, NatalyaTeixeira Guimarães, ClaudiaIvic, IvanaTeixeira, AntonioIwaizumi, Masakazu G.Teixeira, GenerosaIwaniuk, PiotrTeixeira, NatérciaIzquierdo, JordiTeixeira, RóbsonIzydorczyk, GrzegorzTellgren-Roth, ChristianJaafari, AbolfazlTello, Maria LuisaJablan, JasnaTellone, EsterJacobo-Velazquez, DanielTempesta, MariaJacquinet, MarcTemraleeva, AnnaJacygrad, EwelinaTeparić, RenataJadon, Kuldeep SinghTerentyev, Vasily V.Jagosz, BarbaraTermolino, PasqualeJahanbakhshi, AhmadTerrazas, TeresaJahandideh, ForoughTerzaghi, William BryanJahnke, Gizella GyorffyneTerzi, MassimoJailani, Abdul KaderTešić, ŽivoslavJain, Shri MohanTessem, Jeffery S.Jaiswal, AmitTesta, AntoninoJakkula, VinodTezara, WilmerJakobson, LiinaTezuka, TakahiroJakobs-Schönwandt, DesireeThannhauser, Theodore W.Jakubowski, MarcinTheerakulpisut, PiyadaJakubska-Busse, AnnaTheologidis, IoannisJakubus, MonikaThielen, MarcJama-Kmiecik, A.Thiravetyan, PaitipJamil, MuhammadThirugnanasambandam, Prathima PerumalJamnicka, GabrielaThirumalaikumar, Venkatesh P.Jamshidi, SajadThiruvengadam, MuthuJana, LibantováThomas, John C.Janalikova, MagdaThomas, OberhänsliJancsó, MihályThomas, RichardJanczukowicz, WojciechThöming, GundaJanda, KatarzynaThomsen, Mette GoulJaniak, MichalThorlby, Glenn J.Janiszewska, MartaThorp, GrantJankiewicz, UrszulaThum, RyanJankovska-Bortkevič, ElžbietaThussagunpanit, JutipornJanousek, BohuslavTian, ChunjieJansen, StevenTian, LiJara Palacios, María JoséTian, YanpingJaradat, NidalTiberini, AntonioJaramillo-Morales, Osmar AntonioTibpromma, SaowaluckJardim, AlexandreTichaczek-Goska, DorotaJarecki, WacławTidy, AlisonJariene, ElvyraTietel, ZiporaJaroszewska, AnnaTikunov, Yury M.Jarret, RobertTilahun, ShimelesJarugula, SridharTimlin, Dennis J.Jarzebski, MaciejTimm, StefanJasieniuk, MarieTimsina, BinuJasik, JanTimsit, YouriJasser, IwonaTirado, Diego F.Jastrzębska, AnetaTirado-Corbala, RebeccaJastrzębska, MagdalenaTit, DeliaJasutiene, InaTito, AnnalisaJaswal, RajneeshTiwari, ArjunJat, Shankar LalTiwari, KavindraJaved Chaudhary, HassanTiwari, RahulJaved, Muhammad TariqTiwari, SudeepJavier, Raya-GonzálezTiwari, VivekanandJayaraman, JayTiziani, RaphaelJayawardena, RuvishikaTiznado-Hernández, Martín-ErnestoJedrejek, DariuszTkach, NataliaJedrzejczyk, IwonaTkachenko, KirillJeffers, ElizabethTkacz, KarolinaJelaska, SvenTlak Gajger, IvanaJeliazkova, EkaterinaTobe, HiroshiJellali, SalahTobita, HiroyukiJenčo, JaroslavTodaka, DaisukeJensen, KevinToderich, KristinaJeon, Ji-HyeonTodorov, TodorJeon, Sung HoTodorova, Dessislava A.Jeong, Byeong-ryoolTodorova, VelichkaJeong, Dong-HoonTokarz, BarbaraJeong, JeeyonTolrà, RoserJeong, Rae-DongTomasello, AgostinoJeong, Seon-YongTomasello, SalvatoreJeran, NinaTomassoli, LauraJeszka-Skowron, MagdalenaTomaszewski, DominikJevremovic, SladjanaTomazetto, GeizeclerJewell, Jeremy B.Tombesi, SergioJha, GauravTomczak, ArkadiuszJia, QianminTomi, FélixJian, SiyangTomić, AnaJiang, CaifuTomić, HrvojeJiang, DanTomić, SimonidaJiang, GuangdeTonelli, FernandaJiang, GuoxiangTong, HaoJiang, WenzhuTongaonkar, Prasad C.Jiang, YanTony, HaighJiang, YuemingTopoglidis, EmmanuelJianu, CalinTopp, BruceJibran, RubinaToriba, TaiyoJicsinszky, LászlóTorre, Chiara LaJimbo, HaruhikoTorres Vaamonde, José EnriqueJiménez Arellanes, AdelinaTorres, EstanisJiménez Ballesta, RaimundoTorres-Martínez, LorenaJiménez Cantizano, AnaTorrisi, BiagioJiménez Gómez, Pedro A.Torta, LivioJiménez-Araujo, Ana JoséTortosa, GermánJin, JianJunToscano, ValeriaJin, JingboTosheva, Anita G.Jin, NingTóth, BrigittaJin, QingToth, PeterJin, XiaohuaTouraine, BrunoJin, YanTovar-Sánchez, EfraínJing, QiToyoma, TadashiJinn, Tsung-luoTracy, BenjaminJiri, VrbaTran, KimJo, HyunTran, Ngoc NguyenJoão Ramalhosa, MariaTran, Phu-TriJócsák, IldikóTranbarger, Timothy JohnJohannes, WöstemeyerTravaglia, Claudia N.Johnsen, KurtTravlos, IliasJohnson, DonnTrávníček, PavelJohnston-Monje, DavidTrdan, StanislavJokipii-Lukkari, SoileTreder, KrzysztofJokovic, NatasaTrejo-Téllez, Libia IrisJolánkai, MártonTrematerra, PasqualeJones, CharlotteTrendafilova, AntoanetaJones, Meriel G.Trevisan, MarcoJones, MichelleTriacca, UmbertoJones, Richard S.Triantafyllidis, VassiliosJoniec, JolantaTrif, MonicaJordan, Richard W.Trifan, AdrianaJørgensen, KirstenTrifilò, PatriziaJoshi, RohitTrigiano, RobertJotautiene, EgleTrigueros, EsterJozef, MravecTrivellone, ValeriaJózsef, SzatmáriTroganis, AnastassiosJuárez-Maldonado, AntonioTrojak-Goluch, AnnaJubery, Talukder Z.Trontelj, JanezJudita, Zozomová-LihováTrovato, EmanuelaJudzentiene, AstaTrovato, MaurizioJug, DanijelTrucillo, PaoloJuhász, CsabaTrumbić, ŽeljkaJukić, ŽeljkoTrumble, John T.Julca, IreneTruniger, VerónicaJulier, BernadetteTruong, CamilleJung, Ho WonTruong, Hai AnJuráček, MiroslavTruzzi, EleonoraJuranović Cindrić, IvaTruzzi, FrancescaJurasekova, ZuzanaTrzewik, AleksandraJurica, KarloTsaballa, AphroditeJurina, TamaraTsafouros, AthanasiosJurinjak Tusek, AnaTsai, Chen-ChiJurkonienė, SigitaTsai, FumingJusik, SzymonTsai, WenshihJuśkiewicz, JerzyTsakalos, JamesKaasik, HelleTsakiri, EvdoxiaKabala, KatarzynaTsakirpaloglou, NikolaosKabange, Nkulu RollyTsakmakis, Ioannis. D.Kabeya, NaokiTsaniklidis, GeorgiosKačániová, MiroslavaTsarouhas, VasiliosKačergius, AudriusTschaplinski, TimothyKacjan Maršič, NinaTseng, Chih-HuaKadri, AdelTseng, Yu-ChienKadžienė, GražinaTsiaka, ThaliaKafarski, PawelTsikou, DanielaKafel, AlinaTsim, KarlKafle, LekhnathTsioras, PetrosKahraman, KevserTsitsekian, DikranKailasa, Suresh KumarTsouknidas, AlexanderKailis, StanTsubota, HiromiKakabouki, IoannaTsuchihashi, RyotaKalbarczyk, RobertTsui, To-HungKalendar, RuslanTsui, Tsz Kin MartinKalia, Vipin ChandraTsukagoshi, SatoruKalinin, VladimirTsvetov, NikitaKalinova, JanaTsyganov, Viktor E.Kalisz, StanisławTsyganova, Anna V.Kalle, RaivoTucci, MarinaKalloniati, ChrysanthiTucker Lima, Joanna M.Kamal, Abu Hena MostafaTucker, Gordon C.Kamenetsky, RinaTudela, JoseKamińska, MonikaTuenter, EmmyKamiński, DariuszTuleja, MonikaKamisah, YusofTullius Scotti, MarcusKamiya, KoichiTulpova, ZuzanaKamiya, MitsunobuTumbarski, YulianKamjunke, NorbertTünde, JurcaKamran, MuhammadTundis, RosaKandagalla, ShivanandaTung, Chih WeiKandler, WolfgangTungmunnithum, DuangjaiKanetis, LoukasTunon, HakanKang, Ho-MinTuran, MetinKang, In-KyuTurčić, PetraKang, JunmeiTurek, AnnaKang, Kwon KyooTurkiewicz, Igor PiotrKang, NamgooTurmukhametova, NinaKang, Woo HyunTurnau, KatarzynaKang, YoonjaTuroczi, GyorgyKanto, TakeshiTurtoi, MariaKao, Shung-TeTuttolomondo, TeresaKapiel, TarekTvorogova, VarvaraKapusi, EszterTwilley, DanielleKapusta, IreneuszTyagi, Vinay KumarKapusta, PiotrTyburski, JarosławKar, BibekanandaTyburski, JózefKarabagias, IoannisTyler, RobertKaraduman, YaşarTymoszuk, AlicjaKarakaya, AzizTysklind, NiklasKaralija, ErnaTyunin, Alexey PKaram, MohamedTyurin, Alexander A.Karamalakova, YankaTyurin, Anton P.Karamaouna, FilitsaTzanetou, EvangeliaKaranikola, ParaskeviTzen, Jason T.Karantonis, CharalamposTzortzakakis, EmmanouelKarcheva-Bahchevanska, DianaTzortzakis, NikosKargas, GeorgeUberti, FrancescaKarioti, AnastasiaUddin, Md. KamalKaris, Per OlaUdrescu, LucretiaKarkanis, AnestisUgarković, DamirKarki, Subhas S.Uher, DarkoKarlińska, ElżbietaUkalski, KrzysztofKarlov, GennadyUl’yanovskiy, NikolayKarnaouri, AnthiUllah, FazalKaroglan, MarkoUllah, IhsanKarpavičienė, BirutėUllah, NajeebKarpova, Olga V.Ullah, RiazKarsai, IldikoUmar, ShahidKartashov, AlexanderUmehara, MikihisaKarthikeyan, AdhimoolamUndersander, DanKarunarathna, Samantha C.Underwood, WilliamKasahara, Ryushiro D.Ungureanu, CameliaKasianov, Artem S.Ungureanu, NicoletaKaškonienė, VilmaUngurianu, AncaKasowska, DorotaUpadhyay, RakeshKasprzak-Drozd, KamilaUpadhyay, SantoshKasuga, TakaoUr Rehman, Muhammad HabibKaszuba, JoannaUrban, OtmarKatanic, ZoranaUrbaniak, JacekKataoka, HiroyukiUrbanowska, AgnieszkaKatayama, TakeshiUrlić, BranimirKatinakis, PanagiotisUrzua, AlejandroKato, TsuneoUsman, BabarKatoh, HiroshiUsuki, YoshinosukeKatona, KrisztiánUtsumi, YoshinoriKatsenios, NikolaosUygun, SahraKatya, GeorgievaUzelac, BrankaKaundal, AmitaUzoma Asuzu, IsaacKaur, RimaljeetVaccari, MentoreKaushik, PrashantVagelas, IoannisKaushik, RajeevValbon, WilsonKavaliauskas, DariusValcheva, VioletaKavas, MusaValdiviesso, TeresaKavi Kishor, Polavarapu BilhanValencia-Cantero, EduardoKaviani, BehzadValentini, MassimilianoKavvadias, VictorValenzuela, Juan LuisKawakami, SatoshiValero, ManuelKawakami, SusumuVales, IsabelKawamura, AkiraValiunas, DeividasKawamura, FumioValiuskaite, AlmaKawaoka, AkiyoshiValkov, VladimirKaya, ErgunValletta, AlessioKayal, Walid ElValliammai, AlaguvelKayama, MasazumiValverde, ClaudioKayano, Shin-ichiVan Andel, TindeKazachenko, AleksandrVan Deenen, NicoleKazachenko, AlexandrVan Den Berg, CássioKazachenko, AnnaVan Der Maesen, Laurentius Josephus GerardusKazdova, LudmilaVan Der Vossen, HerbertKazimierczak, RenataVan Duijn, Bert Van DuijnKazinczi, GabriellaVan Dyke, MichaelKaźmierczak, AndrzejVan Laere, KatrijnKebede, BerissoVan Loo, MarcelaKeepers, KyleVan Quan, NguyenKeereetaweep, JantanaVan Rhijn, NormanKelebek, HasimVan Sundert, KevinKelleher, ColinVan Tassel, DavidKello, MartinVan Tuyl, JaapKelly, AlisonVan Wuytswinkel, OlivierKenfack, DavidVanavichit, ApichartKennedy, RoyVander Mijnsbrugge, KristineKerchev, PavelVangelis, HarrisKeremane, Manjunath L.Vanhoudt, NathalieKereša, SnježanaVannini, AndreaKereszt, AttilaVantarakis, ApostolosKering, Maru K.Vantarová, KatarínaKerr, PhilipVányolós, AttilaKesawat, Mahipal SinghVanzela, AndréKeswani, ChetanVârban, RodicaKevrešan, ŽarkoVarela, Maria CarolinaKezarooni, ElhamVarela, ZulemaKhalid, Khalid A.Varga, MartinaKhaliluev, Marat R.Vargas-García, María Del CarmenKhallouki, FaridVarkonyi-Gasic, ErikaKhamis, YoussefVasaitis, RimvysKhan, AbbasVasantha-Srinivasan, PrabhakaranKhan, AmanullahVasconcelos, Teresa Maria Pinto Coelho AmadoKhan, ArshadVašek, JakubKhan, AzizVaseva, IrinaKhan, BibiVasilakos, ChristosKhan, HabibVasilescu, Maria MagdalenaKhan, ImranVasileva, VilianaKhan, KasimVasincu, AlexandruKhan, Muhammad AzamVassilakos, NikonKhan, MurtazaVassilev, KirilKhan, NadeemVassileva, ValyaKhan, Nafees A.Vaštakaitė-Kairienė, ViktorijaKhan, NasrullahVašut, RadimKhan, ShahVaux, FelixKhan, Shahid AliVázquez-Espinosa, MercedesKhan, WashimVazquez-Hernandez, MariaKhan, Yusra HabibVaz-Velho, ManuelaKhanday, ImtiyazVeasey, Elizabeth AnnKhanh, Tran DangVeenstra, Anneke A.Khapugin, Anatoliy A.Veeramuthu, DuraipandiyanKhasanov, BulatVeerana, MayuraKhashan, Khawla S.Vega, ClaudiaKhater, HanemVega-Mas, IzargiKhavkin, Emil E.Veiga Junior, Valdir F.Khawar, Khalid MahmoodVek, ViljemKheir, Ahmed M. S.Velada, IsabelKhlestkin, VadimVelena, AstridaKhodaeiaminjan, MortazaVelgosova, OksanaKhoder-Agha, FawziVella, Filomena MonicaKhoo, CheangVellak, KaiKhozin-Goldberg, InnaVellorathekkaepadil, VinodKhruschev, Sergei S.Venanzi, RacheleKhwarahm, Nabaz R.Vendl, TomasKianersi, FarzadVenkidasamy, BaskarKicel, AgnieszkaVennapusa, AmaranathaKidoń, MarcinVerdejo-Lucas, SoledadKiefer, ChristianeVeremeichik, Galina N.Kiefer-Meyer, Marie-ChristineVergara Oñate, Juan JoséKiełkiewicz, MałgorzataVergine, MarziaKiełkowska, AgnieszkaVerissimo, PaulaKiełtyka-Dadasiewicz, AnnaVerma, Akalesh KumarKikuchi, HaruhisaVerma, Praveen C.Kil, Eui-JoonVernet, PhilippeKilanowicz, AnnaVeronico, PasquaKim, ChangsooVerotta, LuisellaKim, Chong-TaiVerrillo, MariavittoriaKim, Chuk YoungVershinin, Alexander V.Kim, ChulminVerstraeten, IngeKim, DaeilVeselova, Svetlana V.Kim, DongGwanVesga, PilarKim, Haeng-hoonViapiana, AgnieszkaKim, Ho BangVicaș, Laura GrațielaKim, Hong-DuckVicente, Filipa A.Kim, Hyun-SoonVicré, MaïtéKim, InyongViczián, OrsolyaKim, Jae-HwanVidal, NievesKim, JaeyoonVidican, RoxanaKim, Jin A.Vidovic, BojanaKim, Jin-ChulVidrih, RajkoKim, Jin-KyungVieira, Maria Lucia CarneiroKim, Jung SunVieira, PauloKim, JunheonVieira, VanessaKim, Ki HyunVielba, Jesús MaríaKim, Kyung-MinVigliante, IvanoKim, Sang-TaeVignini, AriannaKim, Seong-HoonVijan, Loredana ElenaKim, Su MinVijverberg, KittyKim, Sun TaeVikram, PrashantKim, SunahVilatersana, RoserKim, SunghanVílchez, Juan IgnacioKim, Tae KonVilela Oliva, Maria LuizaKim, WookViljevac Vuletić, MarijaKim, Yeon-KiVilla Serrano, MariaKim, Youn-sungVillalva, MarisolKimbaris, AthanasiosVillani, MariacristinaKimeklis, Anastasiia K.Villano, CliziaKincaid, Joshua A.Villanueva, AlvaroKioulos, IliasVillegas, DolorsKippes, NestorVinayaka, Ajjampura C.Kirichenko, TatyanaVincze, EvaKirkman, HughVingolo, Enzo MariaKirschbaum, Daniel SantiagoVining, KellyKiselev, Konstantin V.Vinković, TomislavKiselova, YoanaVinogradov, SzergejKishimoto, KyutaroVinogradova, SvetlanaKishor, D. S.Vinogradova, Yulia K.Kisková, TeréziaViola, StefaniaKiss, JózsefVirdi, KamaldeepKiss, RékaVirjamo, VirpiKissoudis, ChristosVisconti, DonatoKitaeva, AnnaVisioli, GiovannaKizeková, MiriamViškelis, JonasKizheva, YoanaViswanath, Kotapati KasiKizis, DimosthenisVitale, AlessandroKlasa, AndrzejVitale, LucaKlavins, LinardsVitale, Rosa MariaKlčová, LenkaVítámvás, PavelKlein, Joshua D.Vitasović Kosić, IvanaKlepikova, Anna V.Vitelli, JoeKlikocka, HannaVitt, Dale H.Klimowicz, AdamVitti, AntonellaKline, PaulViuda-Martos, ManuelKlooster, Wendy S.Vivithanaporn, PornpunKlucakova, MartinaVivodová, ZuzanaKluczyk, AlicjaVlachonasios, KonstantinosKluz, MaciejVladić, JelenaKnapowski, TomaszVladimirov, VladimirKnežević, DesimirVo, Kieu Thi XuanKnežević, MagdalenaVochita, GabrielaKnupp, GerdVoigt, DagmarKo, Horng-HueyVoiniciuc, CătălinKo, Jae-HeungVoitsekhovskaja, OlgaKo, Swee-SuakVokurka, AlešKoba, TakatoVolk, GayleKobayashi, KoichiVolkov, Roman A.Kobayashi, Natsuko I.Volkov, VadimKobayasi, KazuhiroVolkova, Polina Yu.Koceva Komlenić, DaliborkaVoloudakis, Andreas E.Koch, WojciechVolpert, VitalyKocira, SławomirVončina, DarkoKocsy, GáborVoronkov, AlexanderKocurek, MaciejVrchotová, NadêždaKodad, OssamaVrdoljak Tomaš, AnaKodahl, NeteVries, Jan DeKoebnik, RalfVu, Danh C.Koehler, HartmutVukic, MilenaKohyama, NorikoVuko, ElmaKoim-Puchowska, BeataVuts, JozsefKokaeva, LyudmilaVyhnánek, TomášKokoška, Ladislav EavVysetti, BalaramKoksharova, Olga A.Wachowska, UrszulaKolano, BozenaWadas, WandaKolarcik, VladislavWadl, PhillipKolchanov, Nikolay A.Wadsworth, Richard A.Kolesnikov, Sergey IlyichWagh, SopanKollárová, KarinWahla, SadamKoller-Peroutka, MarianneWaines, GilesKołton, AnnaWakabayashi, Ken-ichiKolukisaoglu, ÜnerWakabayashi, TakatoshiKomarova, Tatiana V.Wakil, WaqasKominko, HalynaWakuliński, WojciechKomives, TamasWaldner, PeterKonarska, AgataWalentowski, HelgeKończak, MagdalenaWalker, Robert J.Kong, HanghuiWalkowiak, SeanKong, LishengWallace, LisaKong, QiushengWalsh, OlgaKonieczynski, PawelWalter, MonikaKononenko, Neonila V.Wanapat, MethaKonôpka, BohdanWanchana, SamartKonopka, IwonaWang, CaiyunKonrad, HeinoWang, ChaominKonstantinov, AlexandrWang, DongdongKönyves, KálmánWang, FayuanKooistra, Wiebe H. C. F.Wang, FengKopeć, PrzemysławWang, GuannanKopjar, MirelaWang, HongjunKopriva, StanislavWang, HonglunKoptur, SuzanneWang, HuanzhongKoramutla, MuraliWang, JianboKordrostami, MojtabaWang, JianmingKordyum, Elizabeth L.Wang, JiaweiKorobova, Alla V.Wang, JiruiKorotkov, KonstantinWang, JunKorshunova, Tat’yanaWang, KaiKorus, JarosławWang, KangxuKosakivska, Iryna V.Wang, KuiwuKosakowska, OlgaWang, LeiKoskela, ElliWang, MingliKosmala, MonikaWang, Myeong-HyeonKosmalski, TomaszWang, PingKosobryukhov, AnatolyWang, QingweiKosobucki, PrzemysławWang, RichardKosová, KláraWang, RufengKostecka-Gugala, AnnaWang, RuoshuiKosterin, OlegWang, ShanliKostić, Aleksandar Ž.Wang, ShengyangKostikova, Vera A.Wang, ShiboKostov, Georgi AtanasovWang, SuKostrakiewicz-Gierałt, KingaWang, XiaokeKostrzewa, MagdalenaWang, XinrongKostyn, KamilWang, XuefeiKotenkova, ElenaWang, YafengKothari, Shanker LalWang, YananKotlowski, RomanWang, YiKotov, Alexey A.Wang, ZhijunKottmann, LorenzWang, ZhoufeiKotynia, AleksandraWang, ZhuangjiKoudounas, KonstantinosWani, ShabirKougioumoutzis, KostasWannicke, NicolaKoukounaras, AthanasiosWard, BrianKourkoumpas, Dimitrios-SotiriosWarguła, ŁukaszKoutsaviti, AikateriniWarner, DouglasKoutsos, ThomasWarzecha, ZygmuntKouvelis, Vassili N.Watanabe, TadashiKovač, ŽarkoWatano, YasuyukiKovačević, StrahinjaWawrzyniak, RafałKováčik, JozefWawrzyńska, AnnaKovács, PéterWayment, DarceyKovtonyuk, Nataliya K.Weaver, DavidKowalczyk, PawełWeaver, MarkKowalczyk, TomaszWeber, MichaelKowalska, AnnaWęglarz, ZenonKowalska, HannaWehr, BernhardKowalska, IwonaWei, DongqingKowalska, JolantaWei, FangKowaluk, GrzegorzWei, HaoKoyama, TomotsuguWei, LiKozai, ToyokiWei, ShanshanKozaki, AkikoWei, WeiKozakiewicz, PawełWeiss, JuliaKozhevnikova, Anna D.Wekesa Kasili, RemmyKozieł, EdmundWen, ChanglongKozłowska, JoannaWeng, XiaoyuKozłowska, MariolaWeryszko-Chmielewska, ElzbietaKozuharova, EkaterinaWesołowski, MarekKozyra, MałgorzataWestphal, AndreasKrabel, DorisWheeler, TerryKraj, WojciechWhite, James F.Kramina, Tatiana E.Wicaksono, AdhityoKrapac, MarinWidelski, JarosławKraska, PiotrWidemann, EmilieKraska, ThorstenWidrlechner, MarkKrasuska, UrszulaWieczorek, DorotaKrause, CorneliaWieczorek, Piotr P.Krauze, MagdalenaWierzbowska, JadwigaKravets, VolodymyyrWiesław, PtachKrawczyk, KrzysztofWiewióra, BarbaraKrczal, GabrieleWijata, Agata M.Kreft, IvanWijayawardene, NalinKremer, DarioWiland-Szymańska, JustynaKrešimir, BošnjakWilczewski, EdwardKreslavski, VladimirWilde, Henry DaytonKretschmer, MatthiasWilken, MarkusKrier, FrançoisWilliams, JamesKrigas, NikosWillow, JonathanKrisch, JuditWiniarczyk, KrystynaKrischik, Vera A.Winkler, AndreasKrishna, RiteshWinkler, JanKrivoruchko, AnastasiiaWińska, KatarzynaKrivtsov, VladimirWirkijowska, AnnaKrizman, MitjaWittkop, BenjaminKrochmal-Marczak, BarbaraWoch, Marcin W.Krofta, KarelWójciak, MagdalenaKról, AngelikaWojcik, DanutaKról, EwelinaWójcik-Gront, ElżbietaKrompiec, StanisławWójcikowska, BarbaraKron, IvanWójcik-Pszczoła, KatarzynaKronberga, ArtaWojdylo, AnetaKroupin, PavelWojtania, AgnieszkaKrueger, RobertWojtasik-Kalinowska, IwonaKrūma, ZandaWojtunik-Kulesza, Karolina A.Krupa, TomaszWojtunik-Kulesza, Karolina AnnaKrupa-Małkiewicz, MarcelinaWojtyla, ŁukaszKruszewska, JoannaWolf, MatthiasKruszka, DariuszWolińska, AgnieszkaKrystyjan, MagdalenaWolna-Maruwka, AgnieszkaKrzebietke, Sławomir JózefWolski, Grzegorz J.Krzepiłko, AnnaWoltering, ErnstKrzymińska, JoannaWong-Bajracharya, JohannaKrzysiak, ZbigniewWoo, Jeong-HwaKrzyżak, JacekWood, Delilah F.Krzyżanowska-Kowalczyk, JustynaWoodhall, JamesKrzyzowski, MichałWoziwoda, BeataKsiężak, JerzyWoźniak, GabrielaKu, Kang-MoWoźniak, MartaKuan, Yen-ChouWróbel, BarbaraKubaa, Raied AbouWróbel-Kwiatkowska, MagdalenaKubala, SzymonWrochna, MariolaKubo, NakaoWu, BinKubo, TomohikoWu, BosenKubon, MaciejWu, Chi-ReiKucbel, StanislavWu, DiKucharczyk, MarekWu, JiandongKucharski, JanWu, JunziKuchuk, MykolaWu, KeqiangKućko, AgataWu, Li-HsinKuczynska, AnettaWu, LijunKuczynski, AnikaWu, LinboKudoyarova, Guzel R.Wu, QiKuhlman, Thomas E.Wu, TingKuhlmann, MarkusWu, YanqiKukina, Tatyana P.Wu, ZufangKuklová, MargitaWydro, UrszulaKulig, BogdanWyszkowska, JadwigaKulig, RyszardWyszkowski, MirosławKulikova, Natalya A.Xia, HuiKull, TiiuXia, KuaifeiKulpa, DanutaXia, ShengqianKulus, DariuszXiao, BaohuaKumar Agrawal, AshishXiao, WeiKumar Pandey, SudhirXie, MinhaoKumar Sethiya, NeerajXie, YiqunKumar Srivastava, RajivXin, ZhanguoKumar, AjayXiong, HaizhengKumar, AnujXiong, JiaKumar, ArvindXu, FangsenKumar, AshwaniXu, FengKumar, DeepakXu, HongheKumar, DhirendraXu, JianpingKumar, DineshXu, JieKumar, JiteshXu, JiemengKumar, ManishXu, LeKumar, ManojXu, LetianKumar, ManuXu, MenglongKumar, NarenderXu, QiangKumar, NitishXu, TaoKumar, PankajXu, YanKumar, PawanXu, YapingKumar, RiteshXu, YiKumar, RohitXu, ZhanyouKumar, ShaileshXuan, Tran DangKumar, ShivendraXue, DaweiKumar, SudheerXue, ShaowuKumar, UttamXue, YongKumar, VijayXynias, IoannisKumar, VishalYadav, AkhileshKumari, ArpnaYadav, Chandra BhanKumari, SafaaYadav, DhananjayKumarihami, H. M. Prathibhani C.Yadav, ShaileshKumawat, Ashok KumarYadavalli, ChandrasekharKuna, LucijaYahagi, TadahiroKundariya, HardikYakhin, OlegKundu, ArijitYamada, MasashiKunieda, TadashiYamada, ToshihikoKunwar, RipuYamada-Ogatta, Sueli FumieKuo, Chan-YenYamamoto, AkihiroKuo, Ping-ChungYamamoto, NaokiKuo, Yen-WenYamaotsu, NoriyukiKupcinskiene, EugenijaYamasaki, HideoKupriyanova, Elena V.Yan, ChonglingKuroda, ChiakiYan, HaidongKuronuma, TakanoriYan, JiyeKurowska, MarzenaYan, ShuoKurzawa, MarzannaYan, TingxiangKusch, StefanYanagisawa, NaokoKushunina, MariaYang, ChuanyuKuśmierz, SebastianYang, FengqingKutchin, AleksandrYang, HuaKu-Vera, Juan CarlosYang, KaiminKuzmanović, LjiljanaYang, LimingKuznetsov, Vladimir V.Yang, MengquanKuznetsova, Natalya A.Yang, Ming-DerKuźniar, AgnieszkaYang, Seung HwanKwasniewska, JolantaYang, ShuyiKwiatkowski, Cezary A.Yang, WenchaoKwiecien, IngaYang, XiKyraleou, MariaYang, XiujuanKyriazopoulos, ApostolosYang, YongilLa Spina, MichelangeloYang, YuLabokas, JuozasYang, ZujunLabrou, NikolaosYao, ChunsuoLabuschagne, MarykeYao, WenjingLacatusu, Anca-RovenaYaoita, YasunoriLachowicz-Wiśniewska, SabinaYarmolinsky, DmitryLācis, GunārsYarosh, Daniel B.Łacka, AgnieszkaYarra, RajeshLacko Bartosova, MagdalenaYasrab, RobailLaColla, GiovanniYasuda, MichikoLadányi, MartaYasuhiro, TomitakaLagos-Kutz, DorisYatoo, AliLaguarda-Miró, NicolásYavitt, JosephLahuta, Lesław BernardYe, FeiLai, Kuei-HungYeh, Ting-fengLaing, William A.Yeoung, Young-RogLakatos, TamásYerka, MelindaLake, LachlanYi, TaoLal, Milan KumarYildirim, ErtanLal, MohanYin, ChuntaoLal, Raj KishoriYin, XiangLalas, StavrosYin, XuebinLall, NamritaYin, YongtaiLama, KumarYin, ZhiminLamari, FotiniYin, ZhitongLambrev, Petar H.Yli-Halla, MarkkuLambreva, Maya DimovaYokoyama, RyusukeLampayan, RubenYoneyama, KaoriLamponi, StefaniaYoneyama, KoichiLandi, SimoneYoneyama, TadakatsuLanfredi, MariaYong, Miing-TiemLangellotti, Antonio LucaYoon, Hyo InLanoue, JasonYoosefzadeh Najafabadi, MohsenLara, MiguelYordanova, ZhenyaLaranjo, MartaYoshida, Ri-IchiroLarkin, Robert P.You, YangLarran, Alvaro S.Yousefi, MortezaLarrañaga, NereaYoussef, FadiaLarriba Tornel, EduardoYoussef, Helmy M.Larson, Steven R.Yu, Chang YeonLasik-Kurdyś, MałgorzataYu, JialiLastochkina, OksanaYu, JingLászló, BalázsYu, JingweiŁata, BarbaraYu, PujiaLatocha, PiotrYu, QingLatté, Klaus PeterYu, RuitaoLauberts, MarisYuan, JiazhengLaudonia, StefaniaYuan, PeiguoLaura, RossoYuan, QiushengLauria, AntoninoYuann, Jeu-Ming P.Lauriault, LeonardYudina, LyubovLavado, Raúl S.Yurchenko, EkaterinaLavin, MattYuste, JesusLavrenko, SergiyYusuf, Abdullahi AhmedLavric, VasileYusuke, KazamaLaw, WayneZaborowska, MagdalenaLazár, DušanZacchini, MassimoLazarevic, JelicaZada, Elkhan RichardLazaridou, TheanoZadesenets, KiraLazzarato, LorettaZafar, MuhammadLazzaro, LorenzoZafar, ZikriaLe Thanh-Blicharz, JoannaZafrilla, PilarLe, JieZagas, Theocharis D.Le, Nguyen ThanhZagorchev, LyubenLeahu, AnaZagórska-Dziok, MartynaLeandro, SottiliZagoskina, Natalia ViktorovnaLeastro, Mikhail OliveiraZaguła, GrzegorzLe-Deygen, Irina M.Zahra, NoreenLedwożyw-Smoleń, IwonaZaina, GiusiLee, Byung-HyunZaini, PauloLee, Dae-SungZaitsev, GlebLee, HwayongZaitsev, Kirill V.Lee, Hyun-SookZając, AdamLee, Jae HyeokZakharova, Ekaterina V.Lee, Jei-WanZakłos-Szyda, MalgorzataLee, JiyoungZaman, WajidLee, KeunsubZambonelli, AlessandraLee, Kuan-HanZamborine-Nemeth, EvaLee, KwanukZamljen, TilenLee, Louis S. H.Zamulina, InnaLee, Mei-HwaZandstra, BernardLee, MinaZanella, LorenzoLee, RichardZaprutko, LucjuszLee, Sang-HanŻarczyński, PiotrLee, Sang-kyuZargar, MeisamLee, Seung YounZarrelli, ArmandoLee, Sung WooZarrouk, OlfaLee, Tse-MinŻarska, BarbaraLegović, TarzanZarzyńska-Nowak, AleksandraLegrand, SylvainZasada, IngaLegua, PilarZavaleta-Mancera, AraceliLehnert, MarcusZawada, KatarzynaLehnhoff, Erik A.Zdravković Korać, SnežanaLei, XiangZdunek-Zastocka, EdytaLeisegang, KristianŻebrowska, JadwigaLeisova-Svobodova, LeonaZebrowski, JacekLeitão, José M.Zeeb, BarbaraLeitao, LuisZeidler, MiroslavLeitão, Susana TrindadeZekker, IvarLeite, Luan De SouzaZeković, ZoranLeitner, DanielZelena, DoraLejman, AgnieszkaZeljković, Sanja ĆavarLenhard, MichaelZell, RolandLentini, GiovanniZelnik, IgorLenz, VolkerZemlyanskaya, Elena V.Leon, FranciscoZemunik, GrahamLeonelli, GiovanniZeng, JieLeón-Lobos, PedroZeng, LantingLeonova, IrinaZeng, SongjunLeontopoulos, StefanosZerges, WilliamLeport, LaurentZettler, Lawrence W.Lepse, LigaZhai, ZhiyangLepší, MartinZhang, BaogangLes, FranciscoZhang, CankuiLessl, JasonZhang, ChaozhongLeszczuk, AgataZhang, ChengwuLetek, MichalZhang, DapengLevashova, NataliaZhang, ErshuaiLewis Ivey, Melanie L.Zhang, HuitingLexa, MatejZhang, JianLeybourne, DanielZhang, JianboLi, BoZhang, KaiLi, DahongZhang, KerongLi, DemaoZhang, LinLi, GangZhang, LingLi, GenZhang, LuLi, HongweiZhang, MingzhiLi, HuangZhang, PingxianLi, JianboZhang, QiangLi, LiZhang, QianwenLi, LihongZhang, QingweiLi, LingZhang, SaiyangLi, Man-WahZhang, SuiqiLi, MaotengZhang, WeiweiLi, MingzhuoZhang, WenLi, Po-HsienZhang, WenhengLi, QuanqiZhang, WenminLi, RunweiZhang, XiaohuiLi, SimengZhang, XiaolanLi, TianyuZhang, XinzhongLi, WeilinZhang, XiongqingLi, WenwuZhang, XuecaiLi, XiangnanZhang, YongjiangLi, XinigangZhang, YunhuiLi, XiongZhang, ZhenmingLi, YangZhang, ZishanLi, YangruiZhao, ChenchenLi, YinZhao, ChonghangLi, YinxinZhao, HuLi, ZhigangZhao, HuiLi, ZhouZhao, JianfeiLi, ZiminZhao, ShancenLiao, Chien-SenZhao, XiangyuLiao, Jiunn-WangZhao, XianhaiLiao, WeibiaoZhao, XinhuaLiao, ZhihuaZhao, XiyangLiatikus, ŽilvinasZhao, XueqiangLiberacki, DanielZhao, YunLiberati, DarioZhao, ZhenzhenLibiete, ZaneZhao, ZhipengLicciardello, ConcettaZhao, ZifanLidon, FernandoZheleva-Dimitrova, DimitrinaLiersch, AlinaZheng, XuhuiLigaba-Osena, AyalewZheng, YoubinLigrone, RobertoZheng, YuhongLiguori, GiorgiaZhiponova, MiroslavaLim, Jin-HeeZhong, ZengtaoLim, Young SeokZhong, ZhenhuiLima, SerenaZhou, GuoyingLima, VanderleiZhou, HuakunLimami, Anis M.Zhou, JunLimauro, DanilaZhou, LibinLin, ChengtaoZhou, LijuanLin, Chia-MinZhou, MingxiLin, Chih-ChienZhou, RongLin, ChujiaoZhou, TianfeiLin, MengZhou, WeiLin, NaichunZhou, XuerongLin, Rung-TaiZhou, ZhongwuLin, Shu-YenZhu, FeifeiLin, WanrouZhu, JianhuaLin, WeichaoZhu, QianhaoLin, XiaoZhu, WeiLin, YuZhu, XiancanLinaldeddu, Benedetto T.Zhu, YaozhouLinares-Pastén, JavierZhu, YiLincoln, Noa KekuewaZhu, ZongshuaiLindberg, SylviaZhuang, JieLindemann, PeterZhuang, JieyunLindig Cisneros, Roberto AntonioZhuang, JingLindow, Steven E.Zhuang, PingLinkevičius, EdgarasZhuang, ShunyaoLinkiewicz, AnnaZhukov, Vladimir A.Liovic, IvicaZhuo, RenyingLipińska, HalinaZhurikova, ElenaLipsa, Florin DanielŽiarovská, JanaLiran, OdedŽibrat, UrošLisciani, SilviaZieba, MalgorzataLisek, JerzyZielińska, SylwiaLiška, AnitaZimmermann, Sabine DagmarLiszka-Skoczylas, MartaZimowska, BeataLittke, KimZine, HamzaLiu, BenyeZingaretti, Sonia MarliLiu, Bin-BinZiogas, VasileiosLiu, ChaoZipori, IsaacLiu, Cheuk LunZiska, Lewis H.Liu, FeiZitterl-Eglseer, KarinLiu, GangcaiZiyatdinova, GuzelLiu, HaiZjalić, SlavenLiu, HailiZlatkovic, MilicaLiu, Ho YihŻołnierczyk, AnnaLiu, HuaZong, NingLiu, HuajianZorić, LanaLiu, JiakaiZornitsa Ivanova, KaterovaLiu, JianZorrig, WalidLiu, JieZotos, AnastasiosLiu, JingranZou, ChengLiu, JizhanZou, JunjieLiu, LeiZou, XilingLiu, LiezhaoZsigmond, LauraLiu, LiyunZsuzsanna, PluhárLiu, LuZubairova, UlyanaLiu, PengZucconi, LauraLiu, QingtingZuena, MartinaLiu, ShiliangŻuk, MagdalenaLiu, WenZuñiga, GustavoLiu, XiangdongZuo, GuangleiLiu, XingŽupunski, VesnaLiu, Yuan-ShuaiŻurawski, JakubLiu, ZhiyongŻurek, GrzegorzLiu, ZhongjianZuther, EllenLiu, ZunyongŽvingila, DonatasLjubičić, NatašaZwolińska, AgnieszkaLjubojević, MirjanaZych, MarcinLlamas, ÁngelZydlik, ZofiaLlanes, AnaliaZykin, Pavel A.Llewellyn, David


